# Supplementary figures and images for: Structural analysis of M1AP variants associated with severely impaired spermatogenesis causing male infertility
Source: PeerJ. 2022 Mar 21;10:e12947. doi: 10.7717/peerj.12947 (PMC8944341; doi:10.7717/peerj.12947)

**A**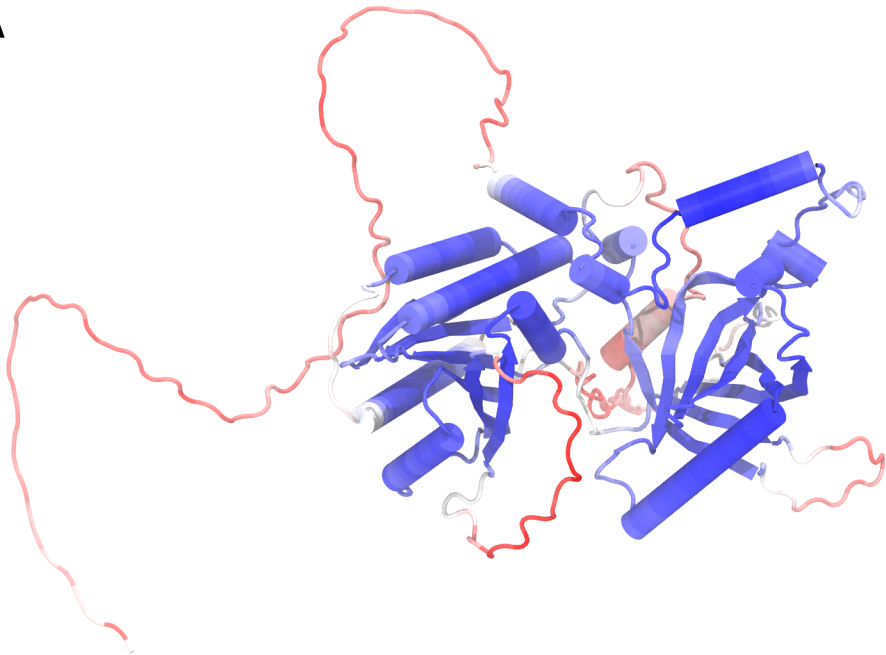**B**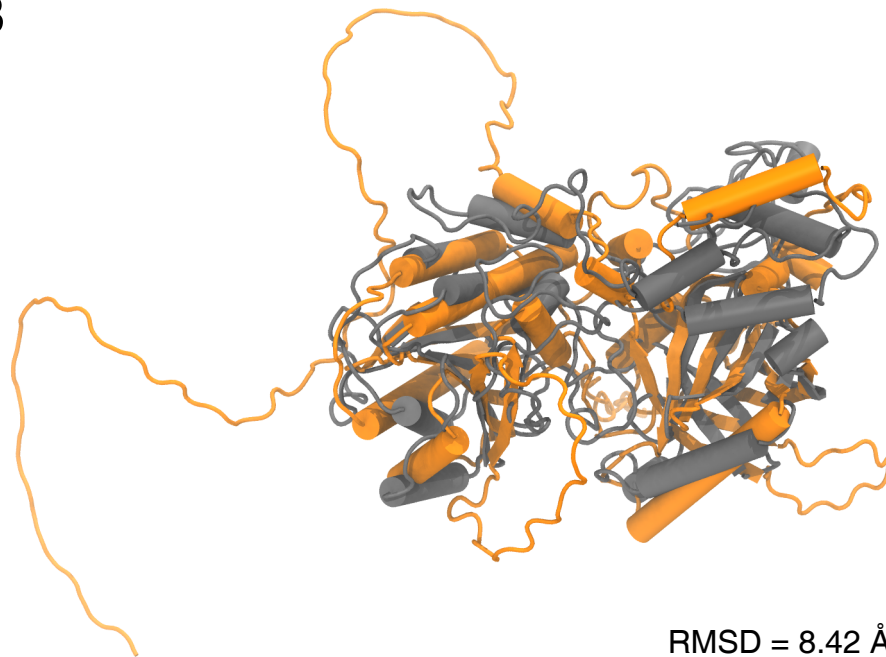

RMSD = 8.42 Å

Supplement: Supplemental Information 1 — (A) M1AP model of AF2. Blue color refers to ≥70% confidence while others indicate lower confidence. (B) Structural alignment of GalaxyWeb (grey) and AF2 (orange) M1AP models, including root-mean-square deviation (RMSD) value. [file peerj-10-12947-s001.pdf]

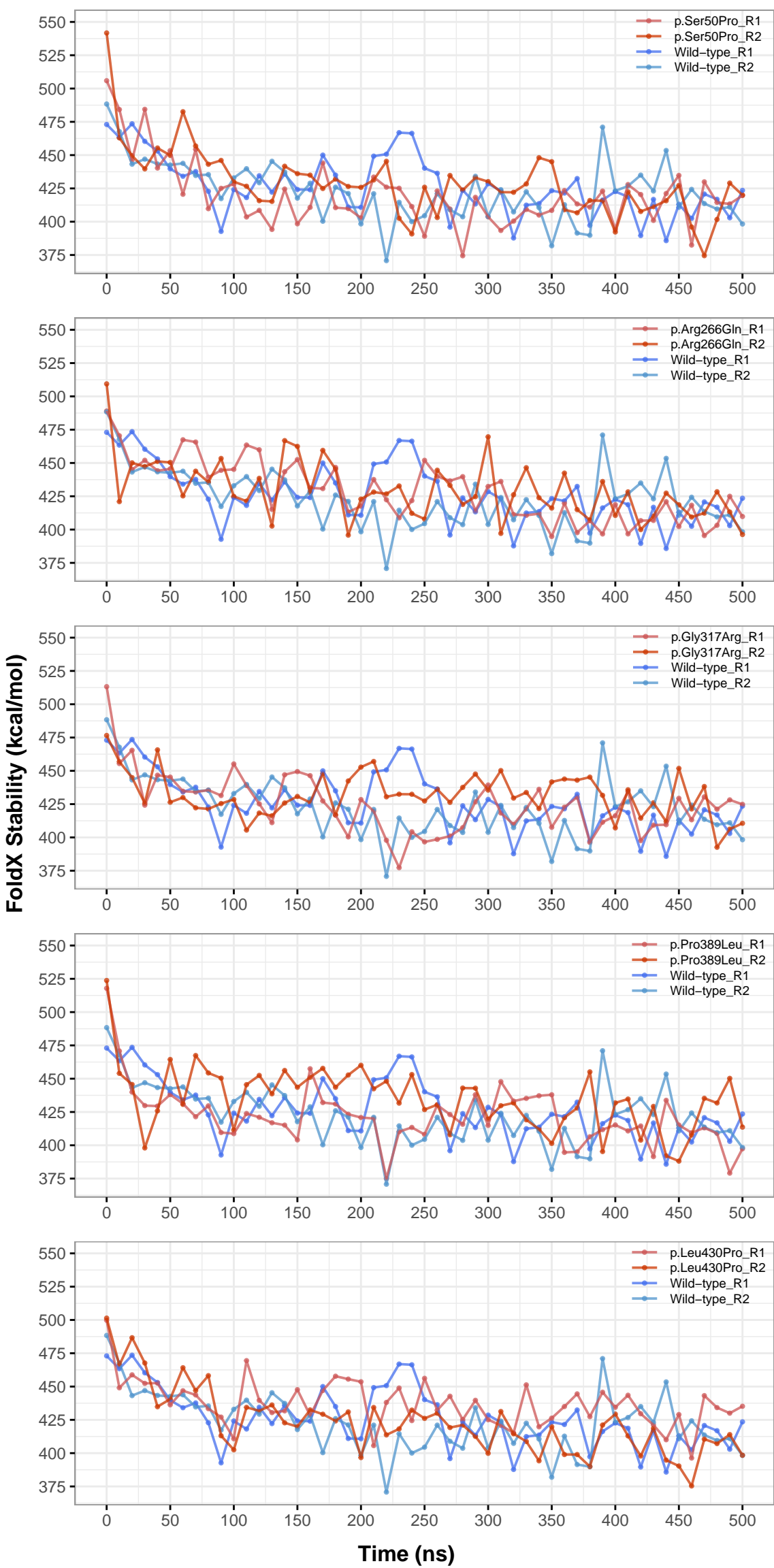

Supplement: Supplemental Information 5 — Stability comparison between molecular dynamics simulations of wild-type and changed M1AP structures. R1 and R2 indicates “Repeat 1” and “Repeat 2” trajectories, respectively. [file peerj-10-12947-s005.pdf]

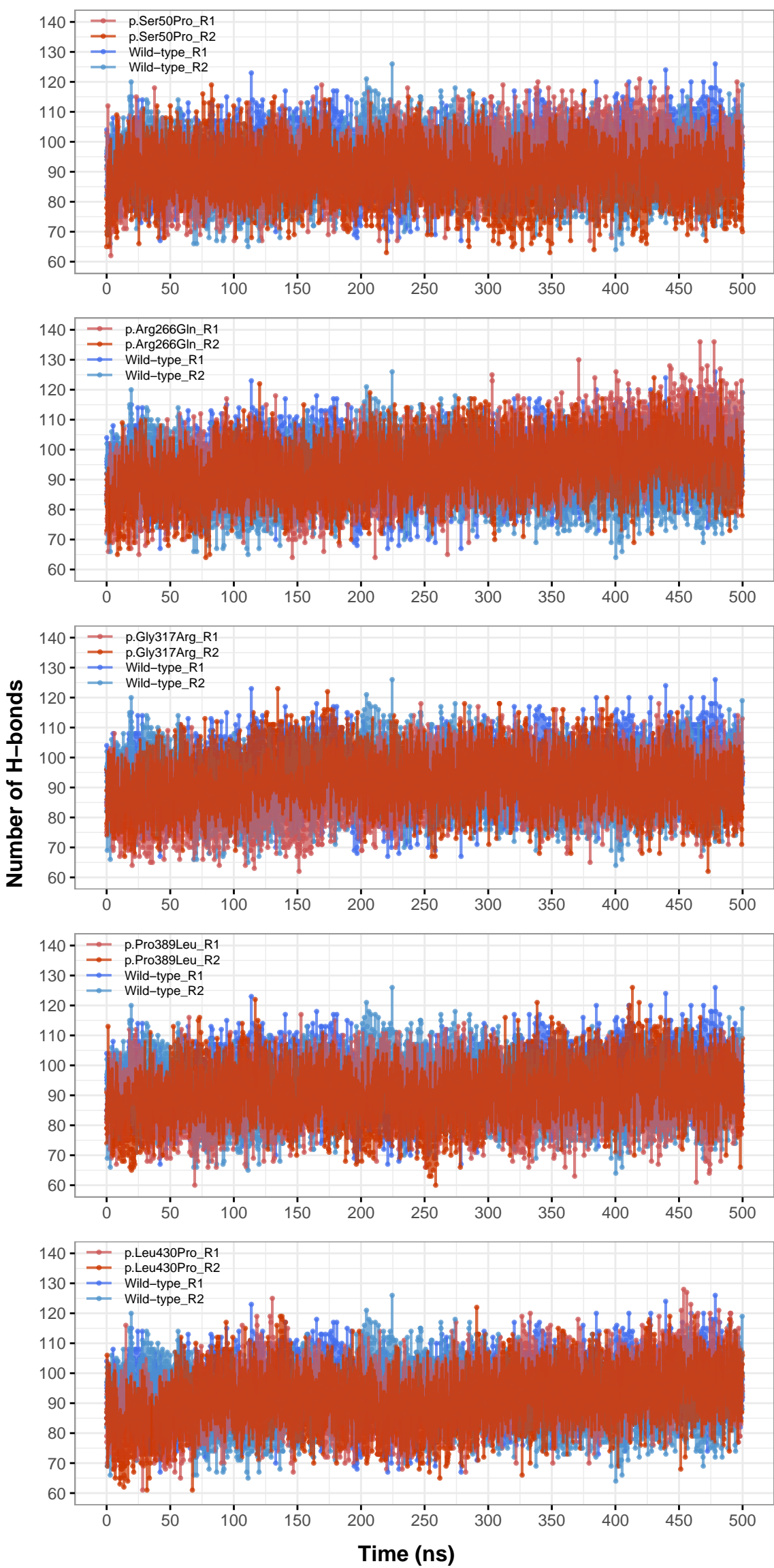

Supplement: Supplemental Information 6 — Comparison of the number of all H-bonds between molecular dynamics simulations of wild-type and changed M1AP structures. R1 and R2 indicates “Repeat 1” and “Repeat 2” trajectories, respectively. [file peerj-10-12947-s006.pdf]

Number of Local H-bonds

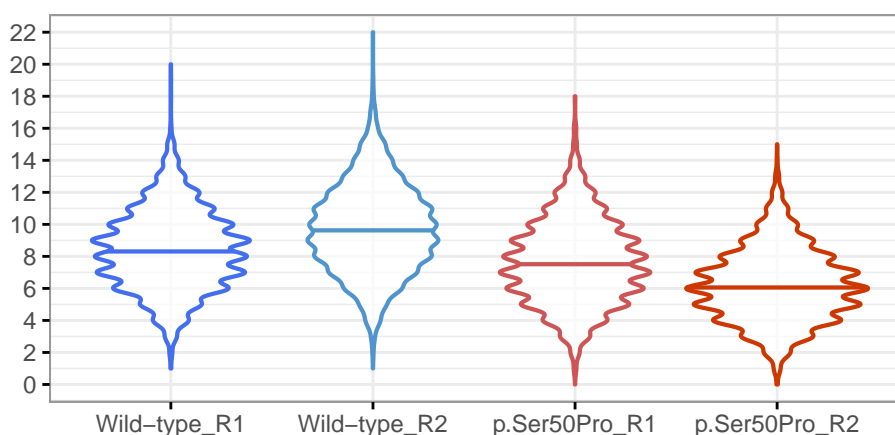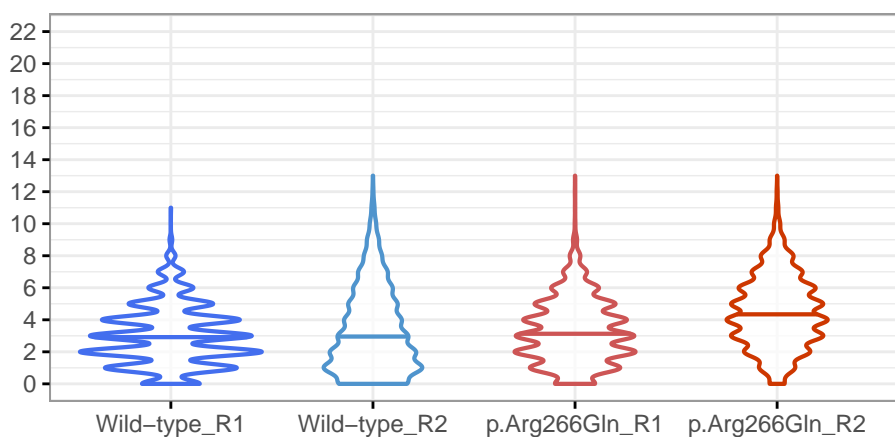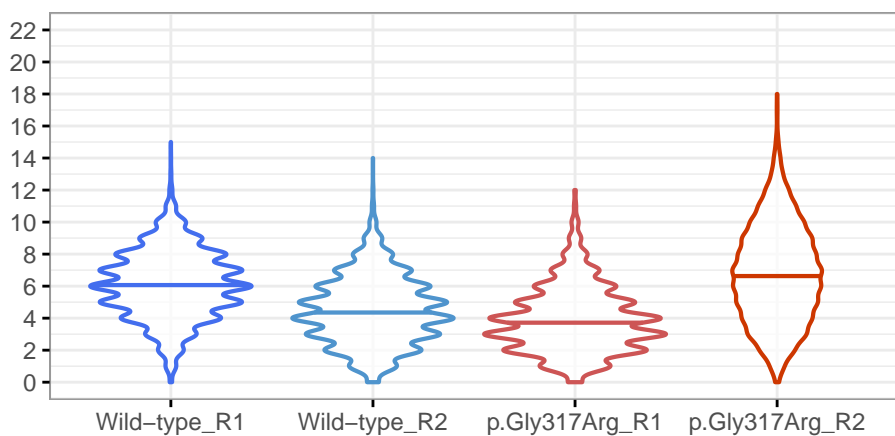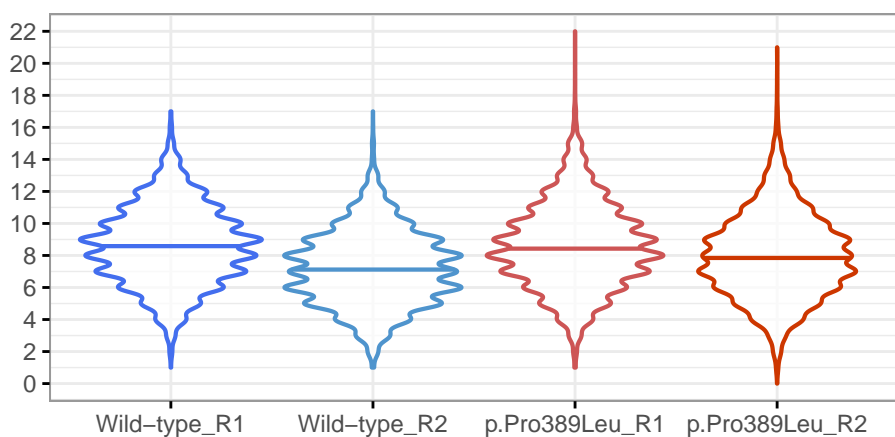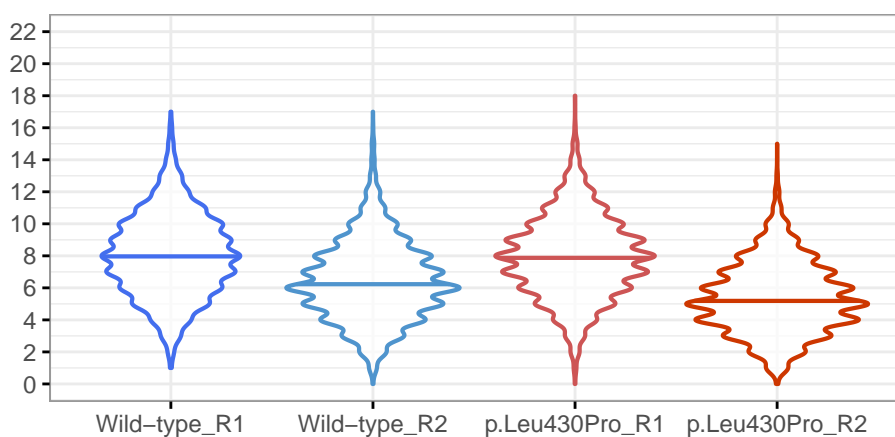

Time (ns)

Supplement: Supplemental Information 7 — Comparison of the number of local H-bonds between molecular dynamics simulations of wild-type and changed M1AP structures. R1 and R2 indicates “Repeat 1” and “Repeat 2” trajectories, respectively. [file peerj-10-12947-s007.pdf]

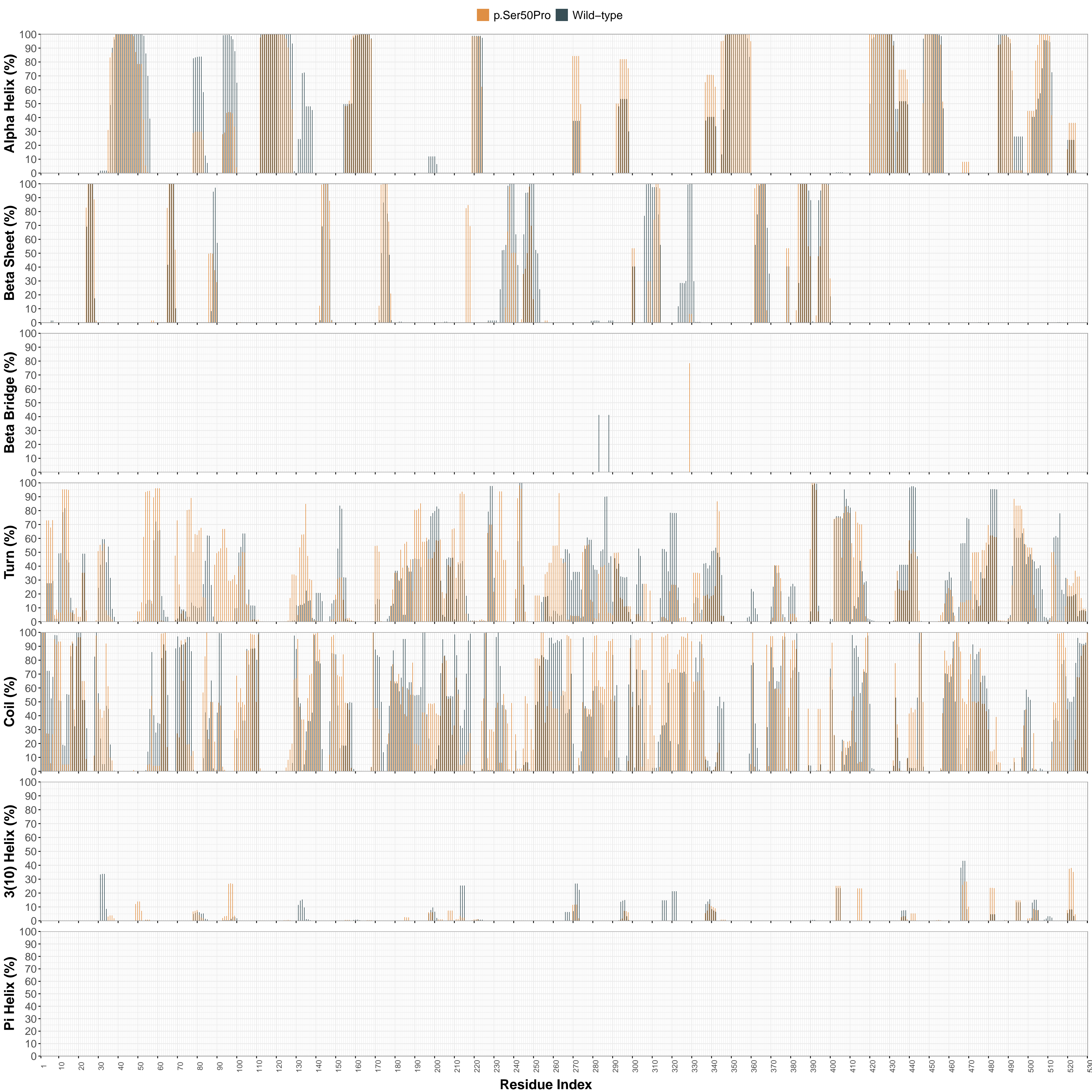

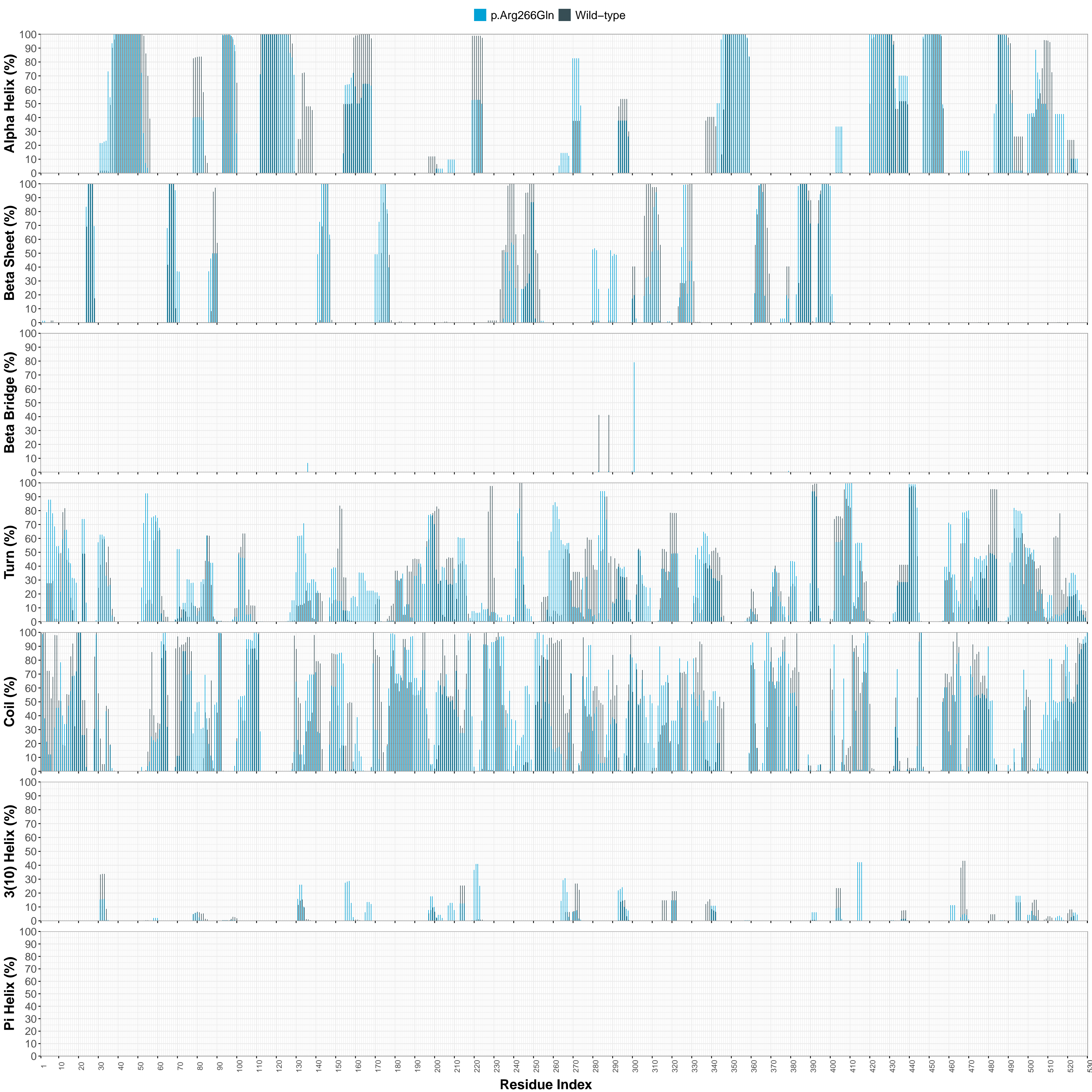

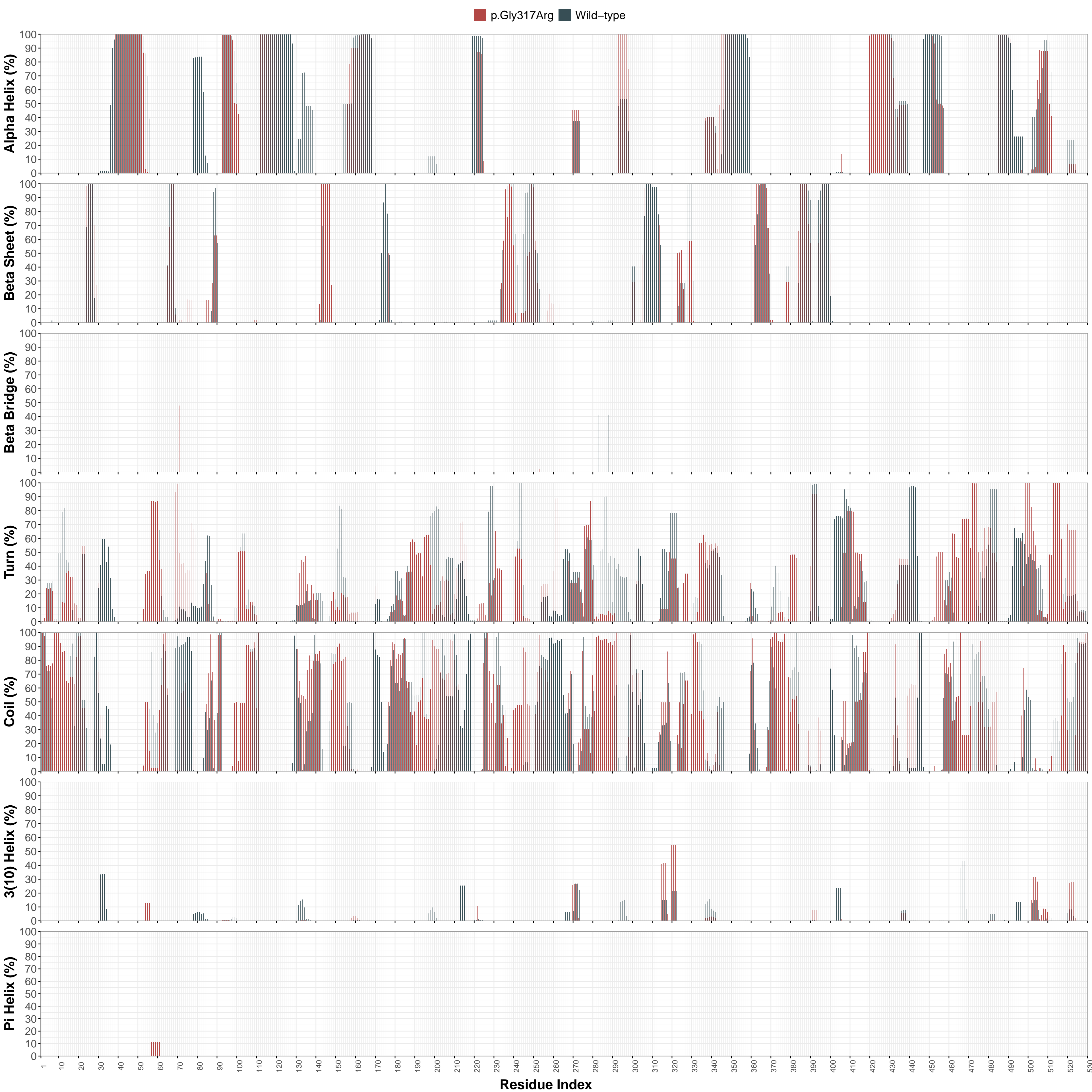

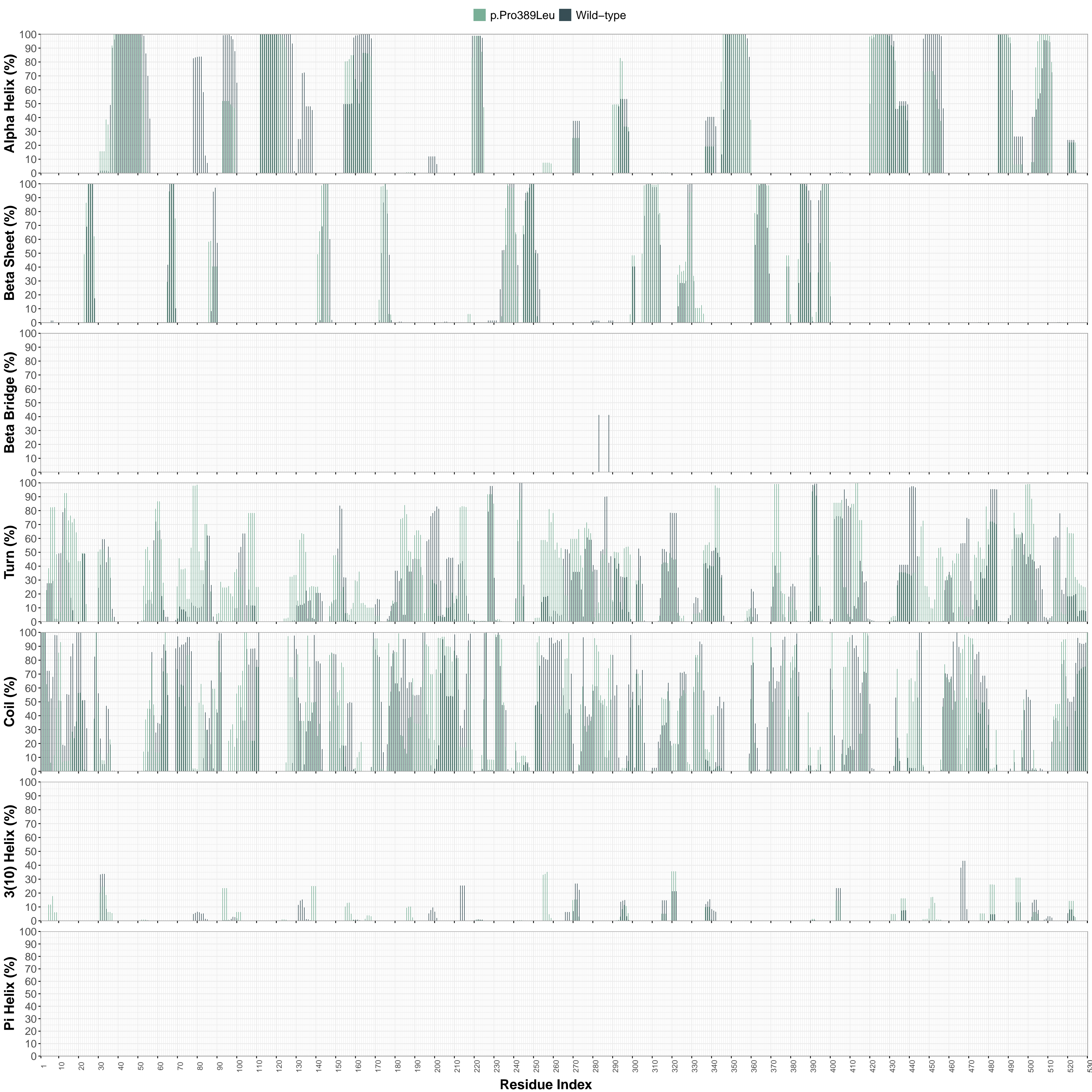

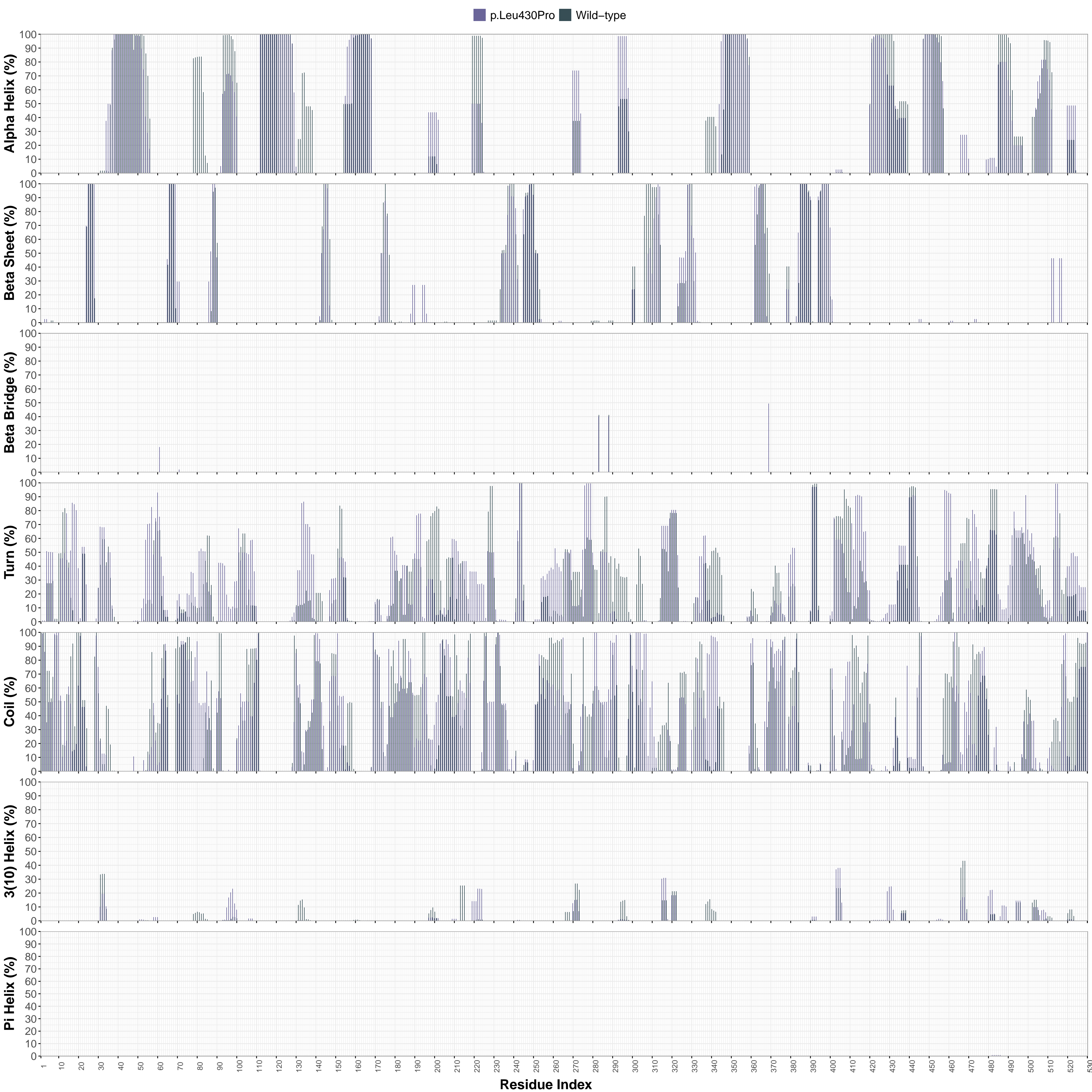

Supplement: Supplemental Information 9 — Secondary structure comparison between molecular dynamics simulations of wild-type and changed M1AP structures. Percentage refers to the percentage of the last 75 ns of simulation during which the amino acids were adopting each one of the secondary structure conformations. [file peerj-10-12947-s009.pdf]
