# Supplementary material for: Structural analysis of M1AP variants associated with severely impaired spermatogenesis causing male infertility
Source: PeerJ. 2022 Mar 21;10:e12947. doi: 10.7717/peerj.12947 (PMC8944341; doi:10.7717/peerj.12947)
Supplement: Supplemental Information 2 — Root-mean-square deviation (RMSD) values are included. Grey color indicate model 1. [file peerj-10-12947-s002.pdf]

GalaxyWeb Models

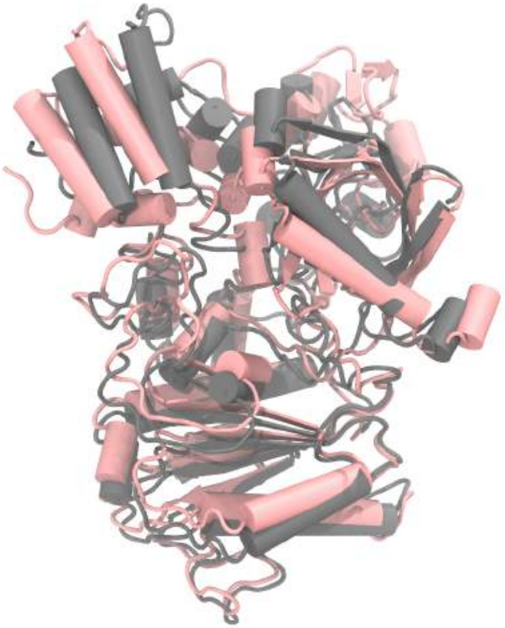

Model 1 vs Model 2
RMSD = 3.01 Á

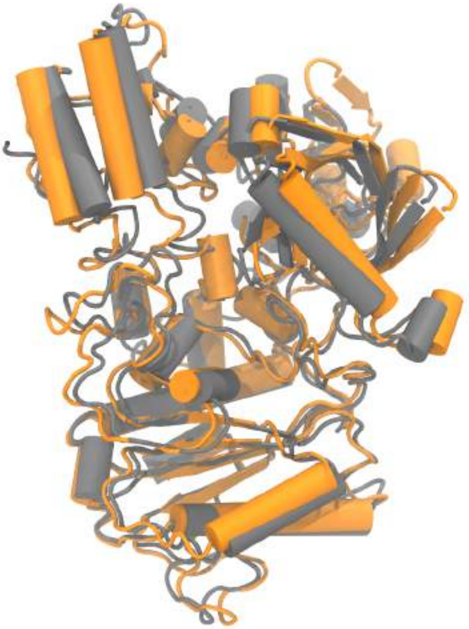

Model 1 vs Model 3
RMSD = 2.05 Á

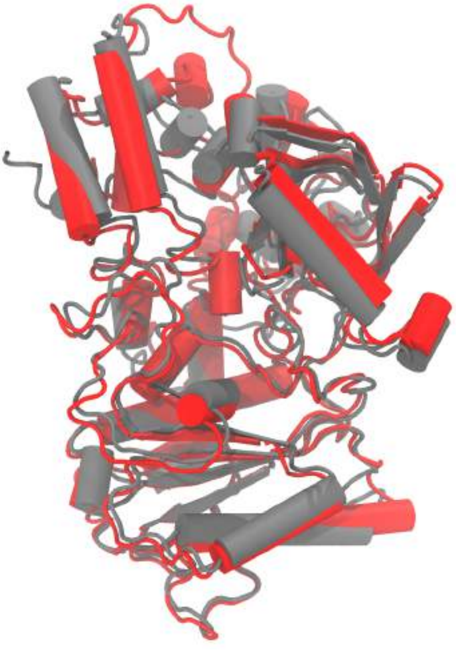

Model 1 vs Model 4
RMSD = 1.76 Á

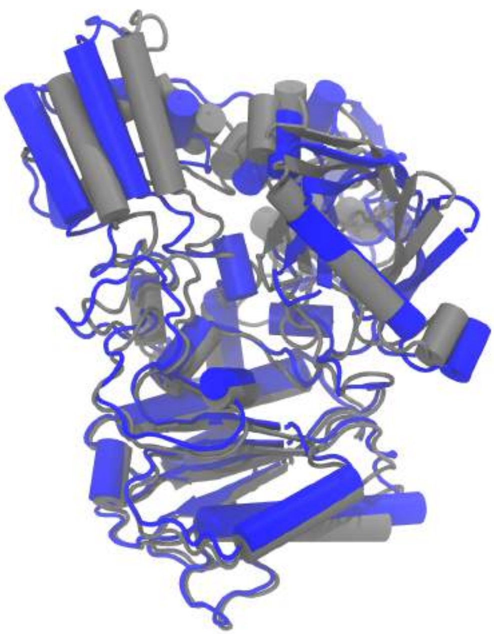

Model 1 vs Model 5
RMSD = 3.28 Á

I-TASSER Models

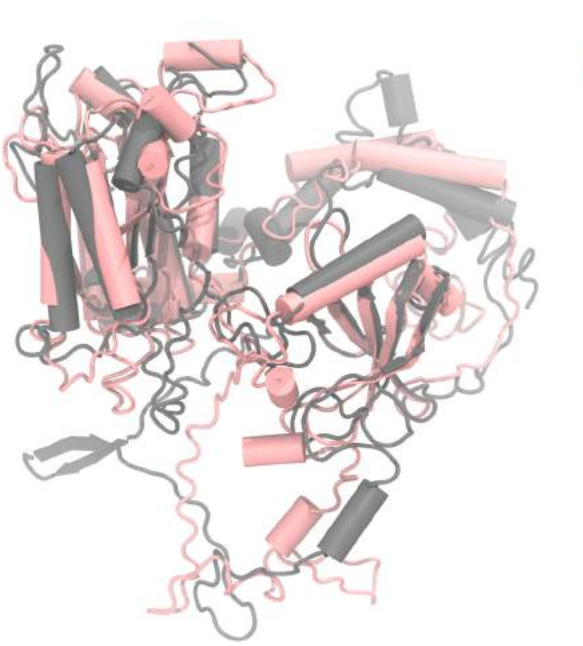

Model 1 vs Model 2
RMSD = 5.43 Á

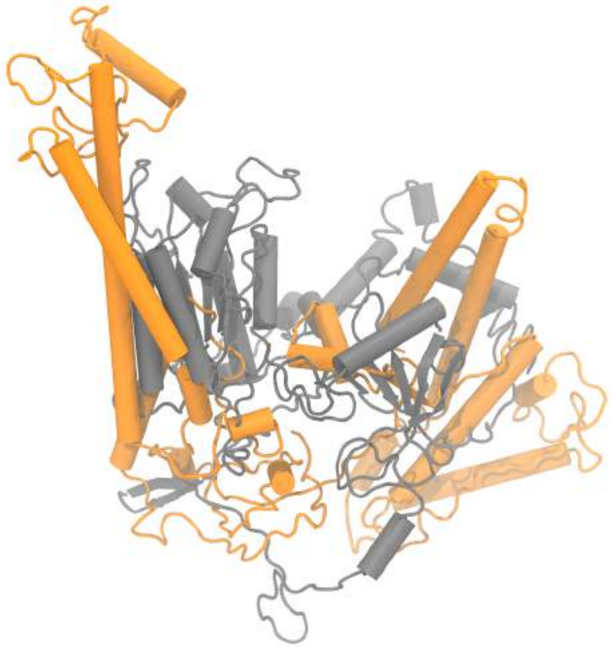

Model 1 vs Model 3
RMSD = 34.33 Á

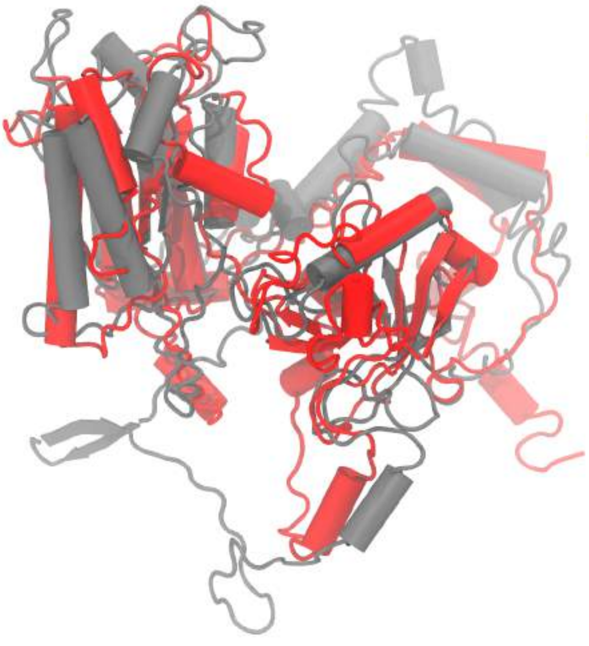

Model 1 vs Model 4
RMSD = 5.66 Á

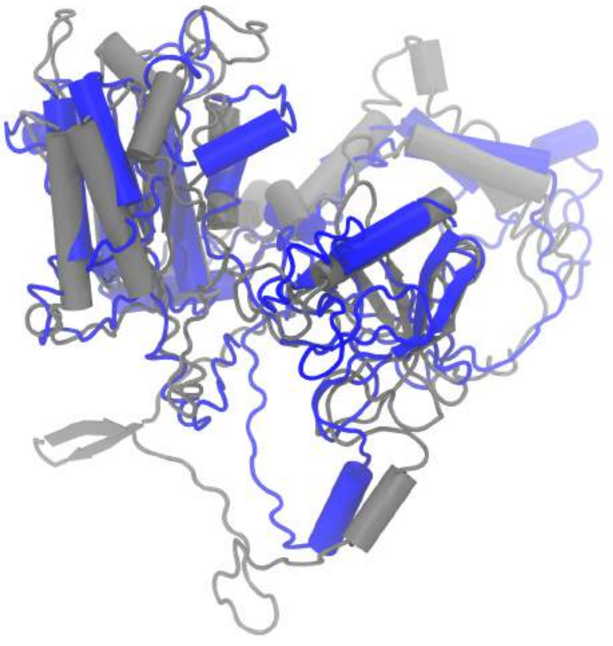

Model 1 vs Model 5
RMSD = 7.18 Á

PRIMO Models

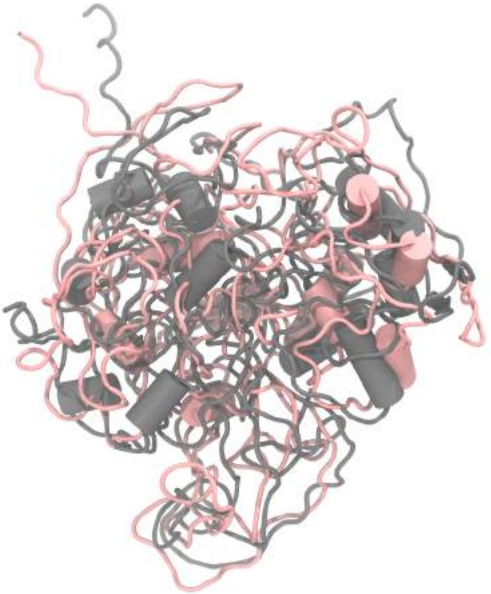

Model 1 vs Model 2
RMSD = 6.79 Á

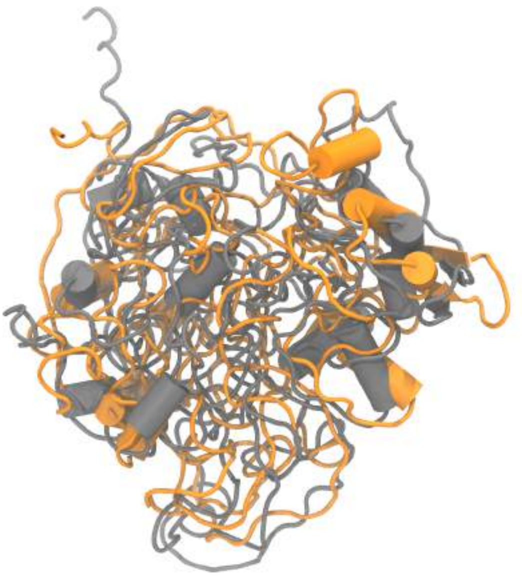

Model 1 vs Model 3
RMSD = 6.44 Á

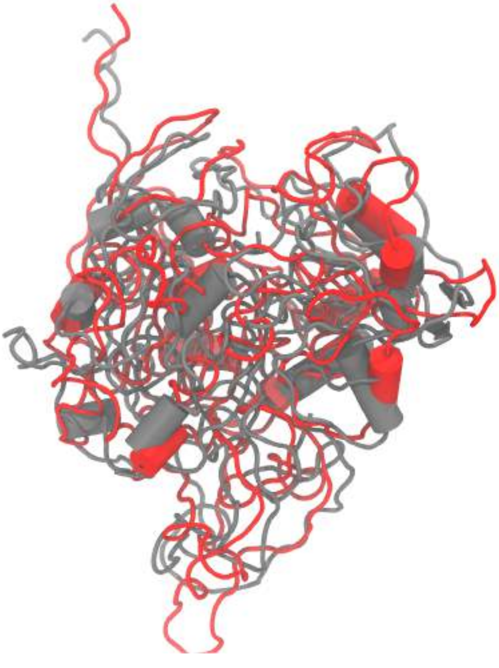

Model 1 vs Model 4
RMSD = 7.13 Á

Robetta Models

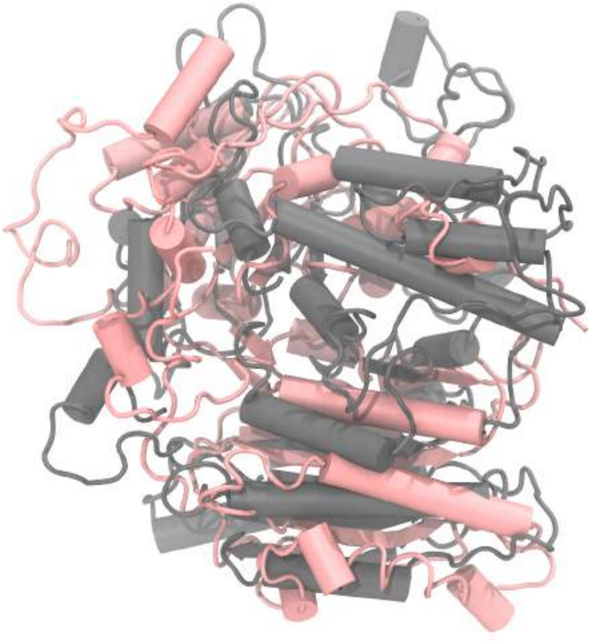

Model 1 vs Model 2
RMSD = 17.91 Á

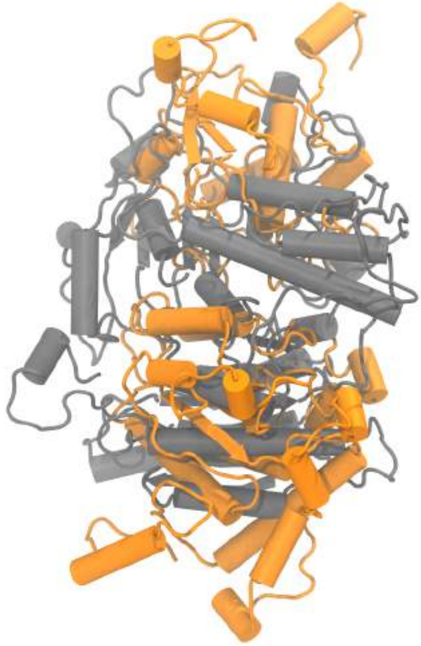

Model 1 vs Model 3
RMSD = 26.94 Á

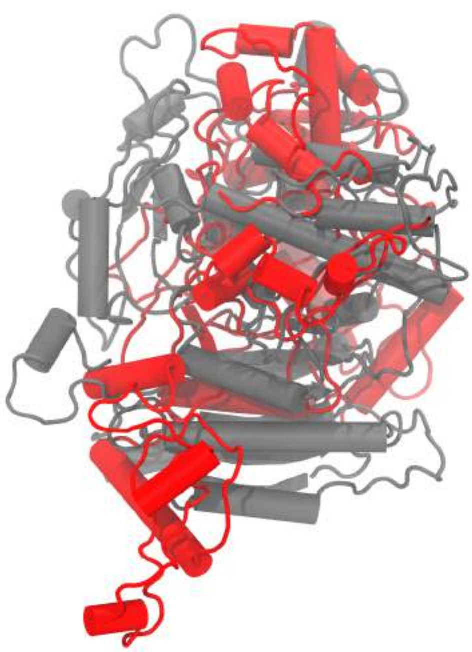

Model 1 vs Model 4
RMSD = 31.50 Á

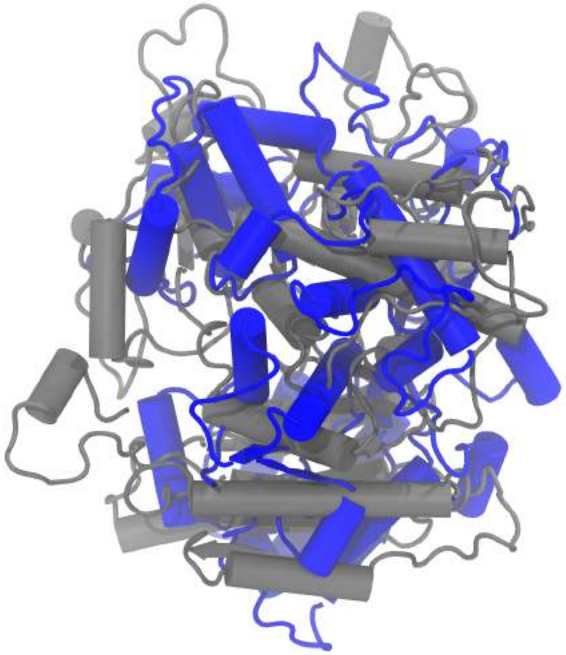

Model 1 vs Model 5
RMSD = 22.95 Á

Robetta-domain Models

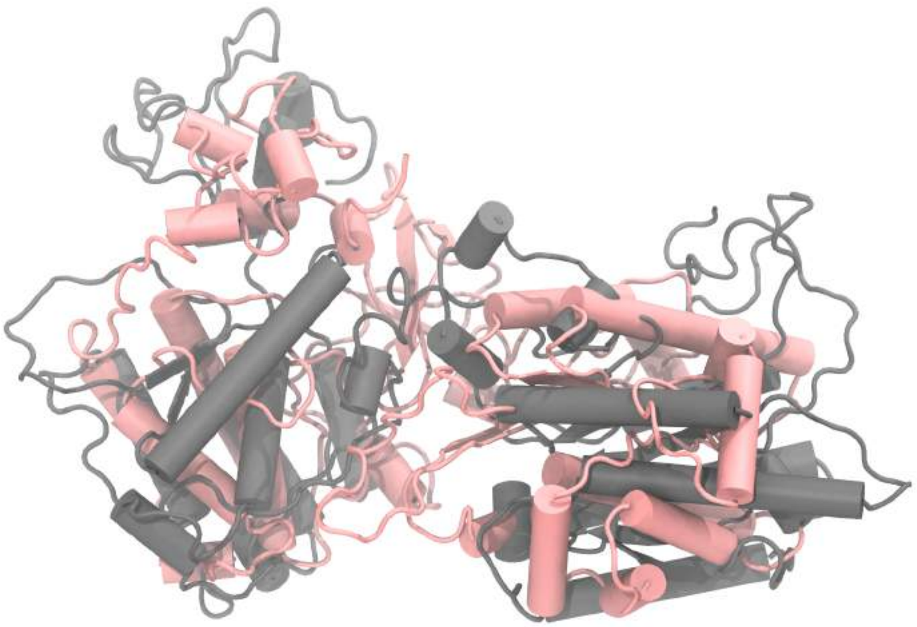

Model 1 vs Model 2
RMSD = 22.40 Á

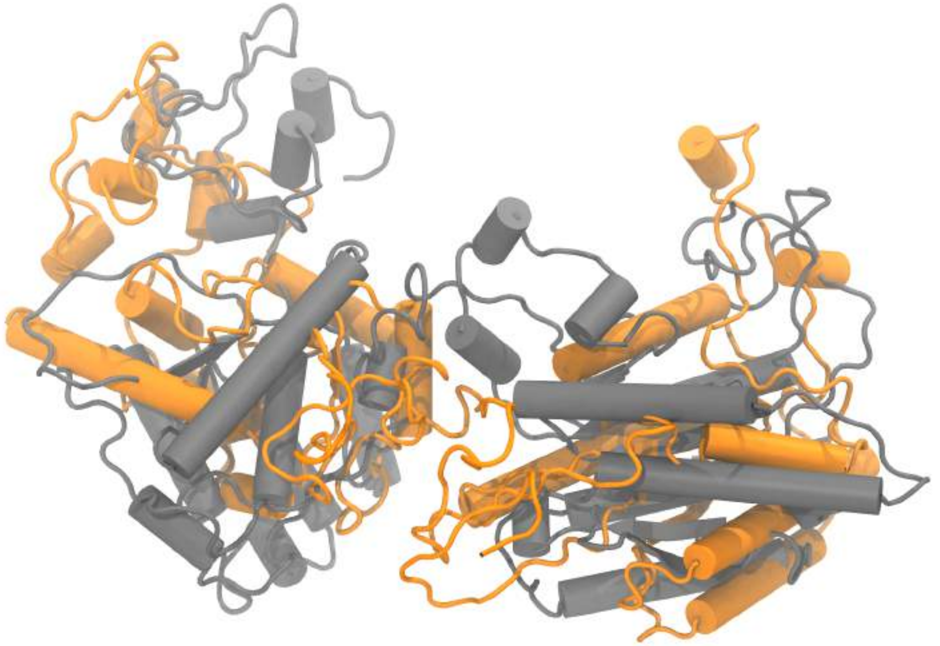

Model 1 vs Model 3
RMSD = 24.88 Á

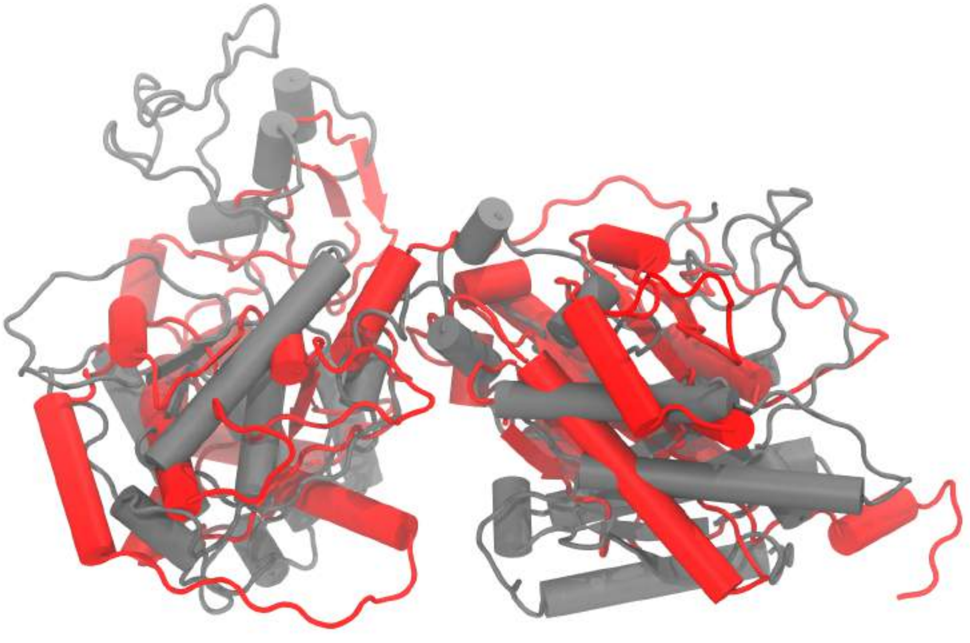

Model 1 vs Model 4
RMSD = 20.35 Á

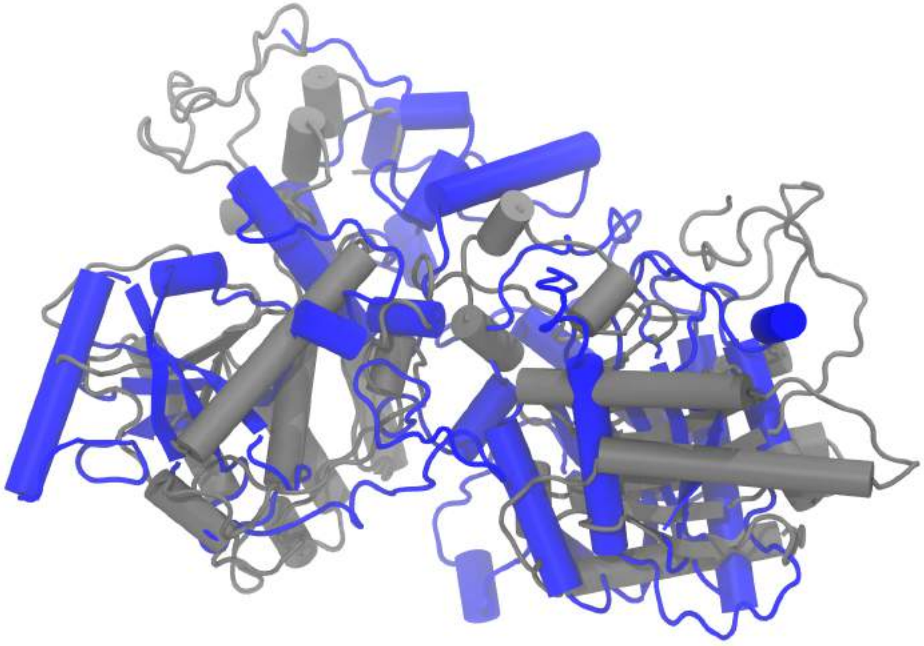

Model 1 vs Model 5
RMSD = 25.38 Á
