# Supplementary material for: Structural analysis of M1AP variants associated with severely impaired spermatogenesis causing male infertility
Source: PeerJ. 2022 Mar 21;10:e12947. doi: 10.7717/peerj.12947 (PMC8944341; doi:10.7717/peerj.12947)

Wild−type

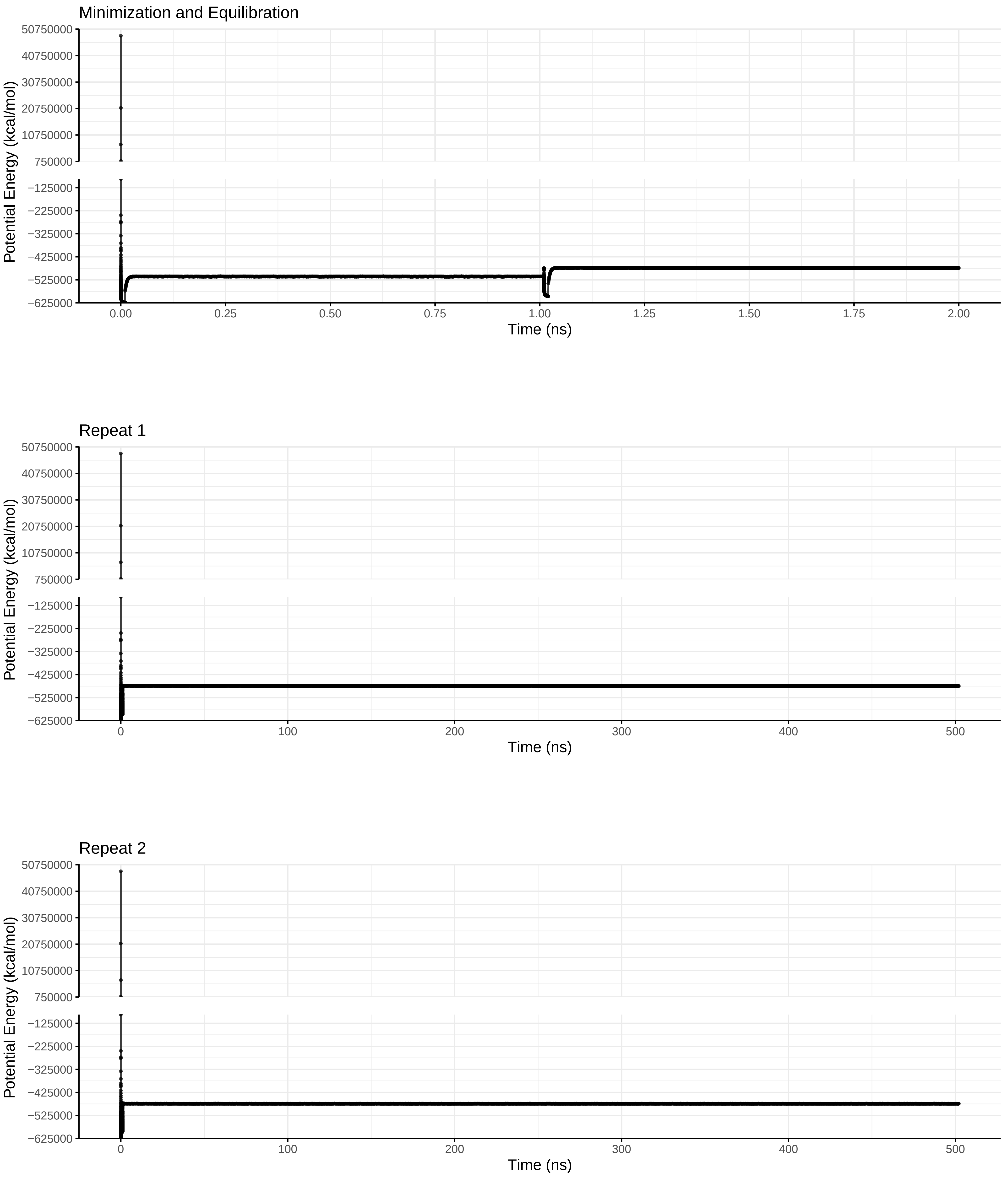

Minimization and Equilibration
Potential Energy (kcal/mol)
50750000
40750000
30750000
20750000
10750000
750000
−125000
−225000
−325000
−425000
−525000
−625000
0.00
0.25
0.50
0.75
1.00
1.25
1.50
1.75
2.00
Time (ns)
Repeat 1
Potential Energy (kcal/mol)
0
100
200
300
400
500
Time (ns)
Repeat 2
Potential Energy (kcal/mol)
0
100
200
300
400
500
Time (ns)

p.Ser50Pro

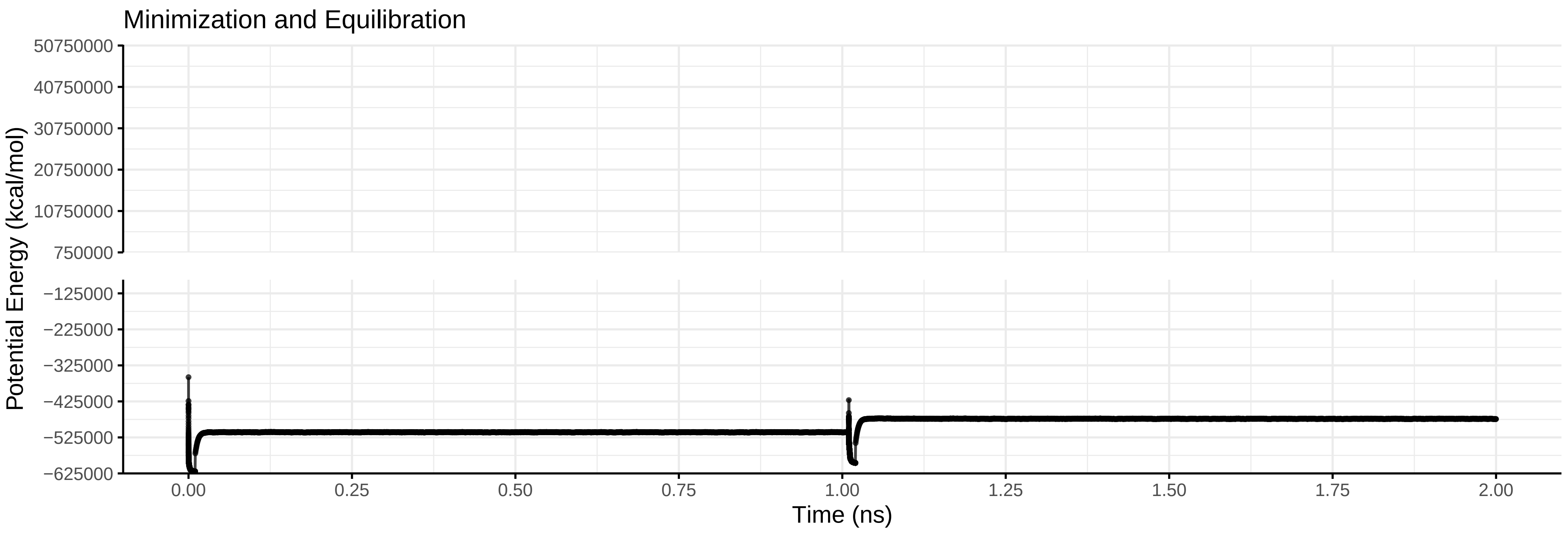
Minimization and Equilibration
Potential Energy (kcal/mol)
50750000
40750000
30750000
20750000
10750000
750000
−125000
−225000
−325000
−425000
−525000
−625000
0.00
0.25
0.50
0.75
1.00
1.25
1.50
1.75
2.00
Time (ns)

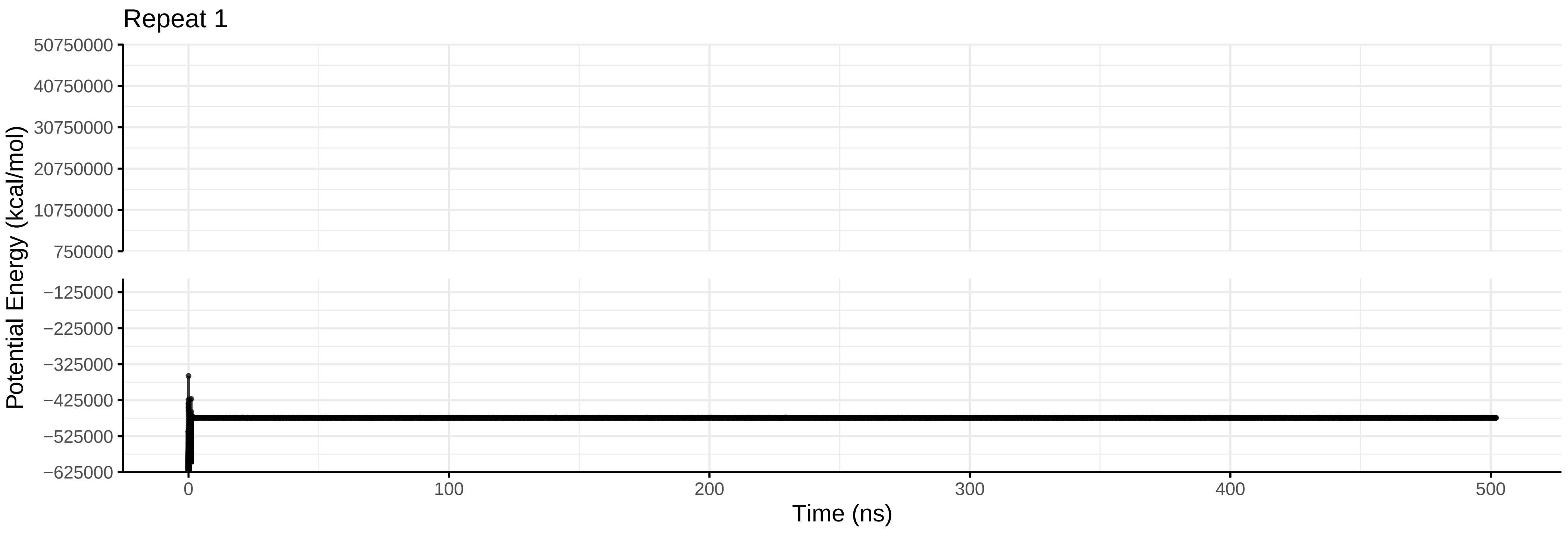
Repeat 1
Potential Energy (kcal/mol)
50750000
40750000
30750000
20750000
10750000
750000
−125000
−225000
−325000
−425000
−525000
−625000
0
100
200
300
400
500
Time (ns)

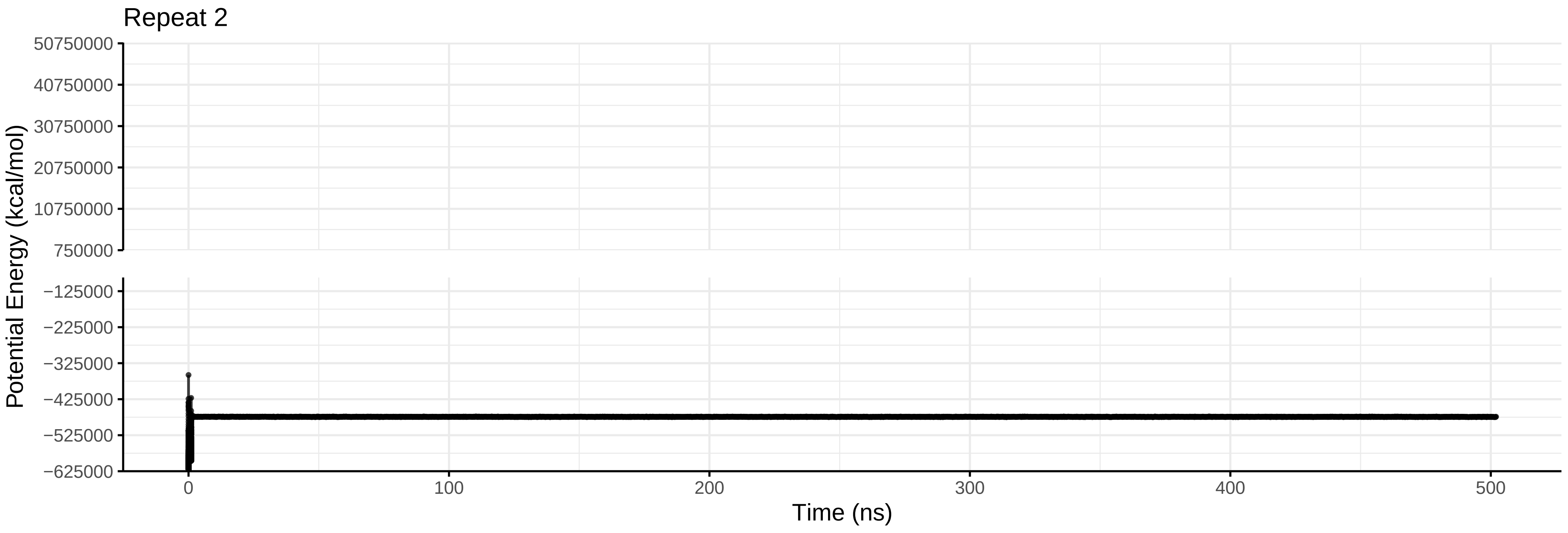
Repeat 2
Potential Energy (kcal/mol)
50750000
40750000
30750000
20750000
10750000
750000
−125000
−225000
−325000
−425000
−525000
−625000
0
100
200
300
400
500
Time (ns)

p.Arg266Gln

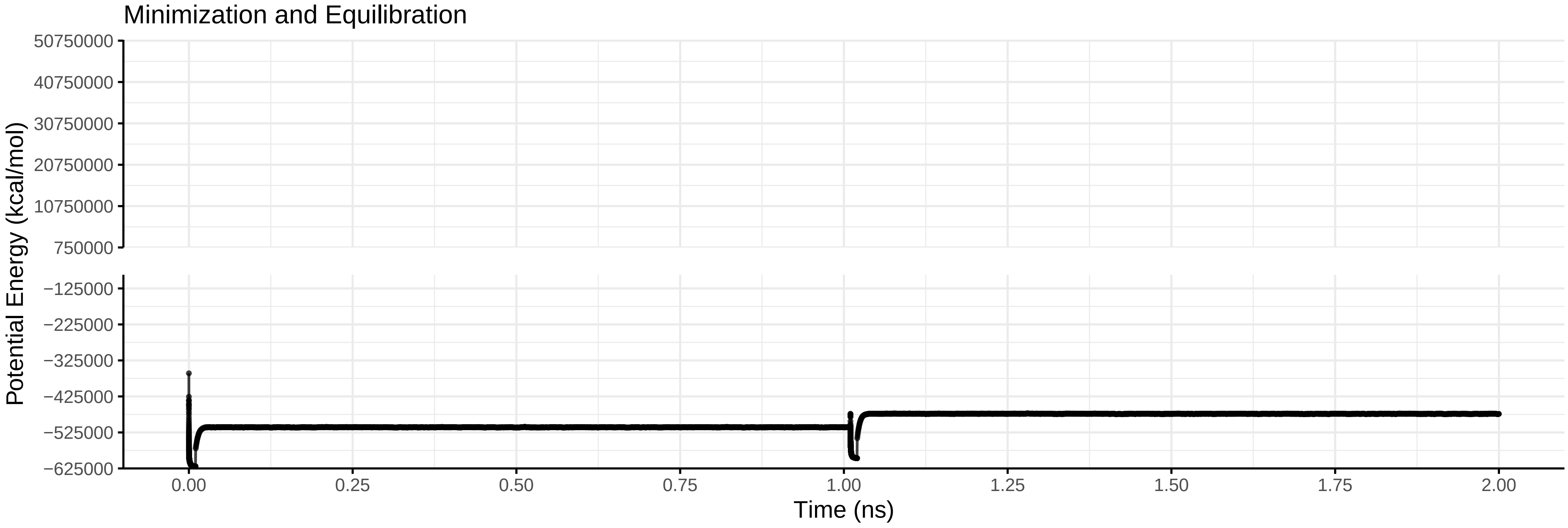
Minimization and Equilibration
Potential Energy (kcal/mol)
50750000
40750000
30750000
20750000
10750000
750000
−125000
−225000
−325000
−425000
−525000
−625000
0.00
0.25
0.50
0.75
1.00
1.25
1.50
1.75
2.00
Time (ns)

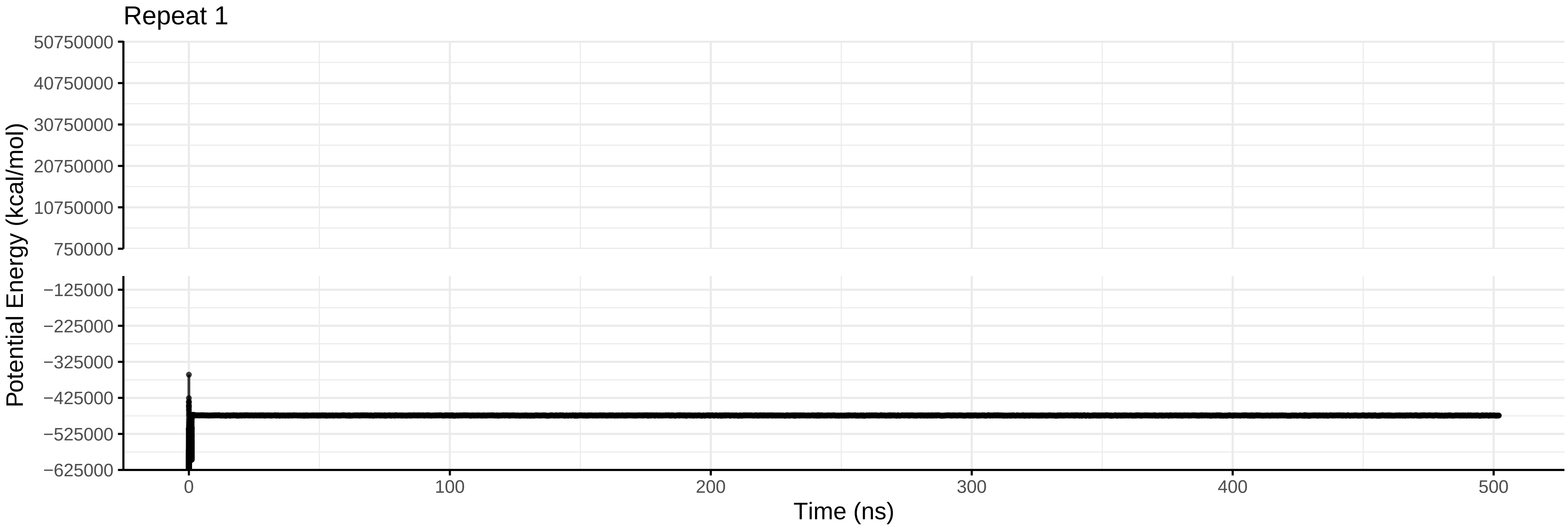
Repeat 1
Potential Energy (kcal/mol)
50750000
40750000
30750000
20750000
10750000
750000
−125000
−225000
−325000
−425000
−525000
−625000
0
100
200
300
400
500
Time (ns)

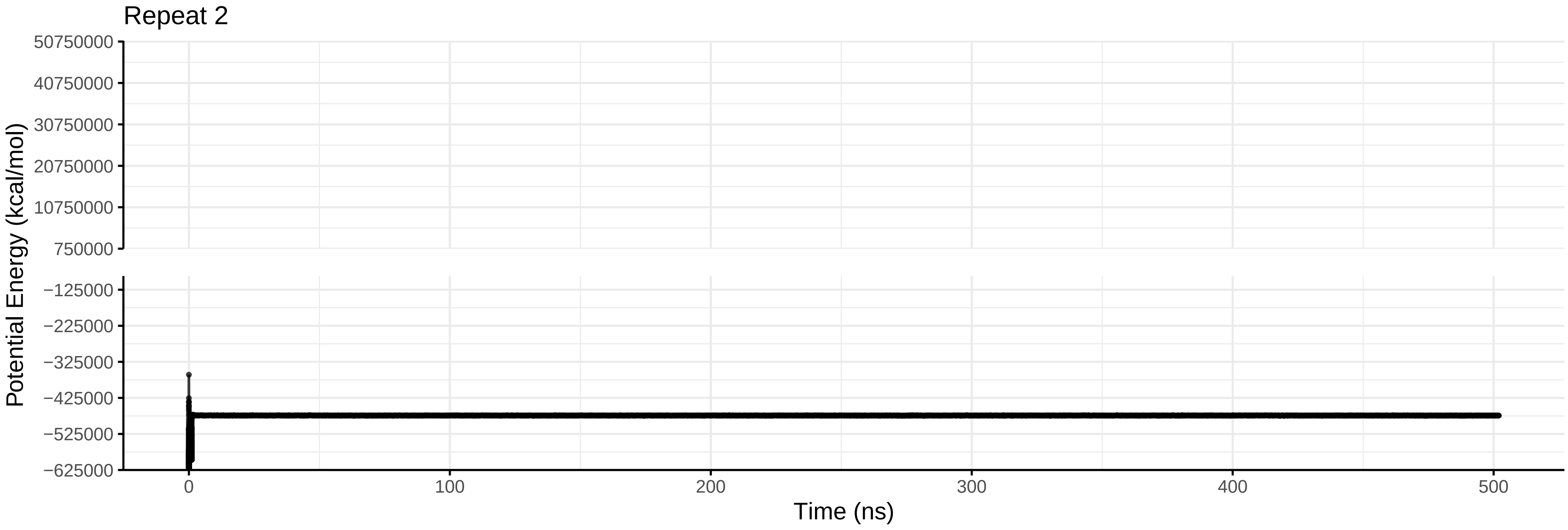
Repeat 2
Potential Energy (kcal/mol)
50750000
40750000
30750000
20750000
10750000
750000
−125000
−225000
−325000
−425000
−525000
−625000
0
100
200
300
400
500
Time (ns)

p.Gly317Arg

Minimization and Equilibration

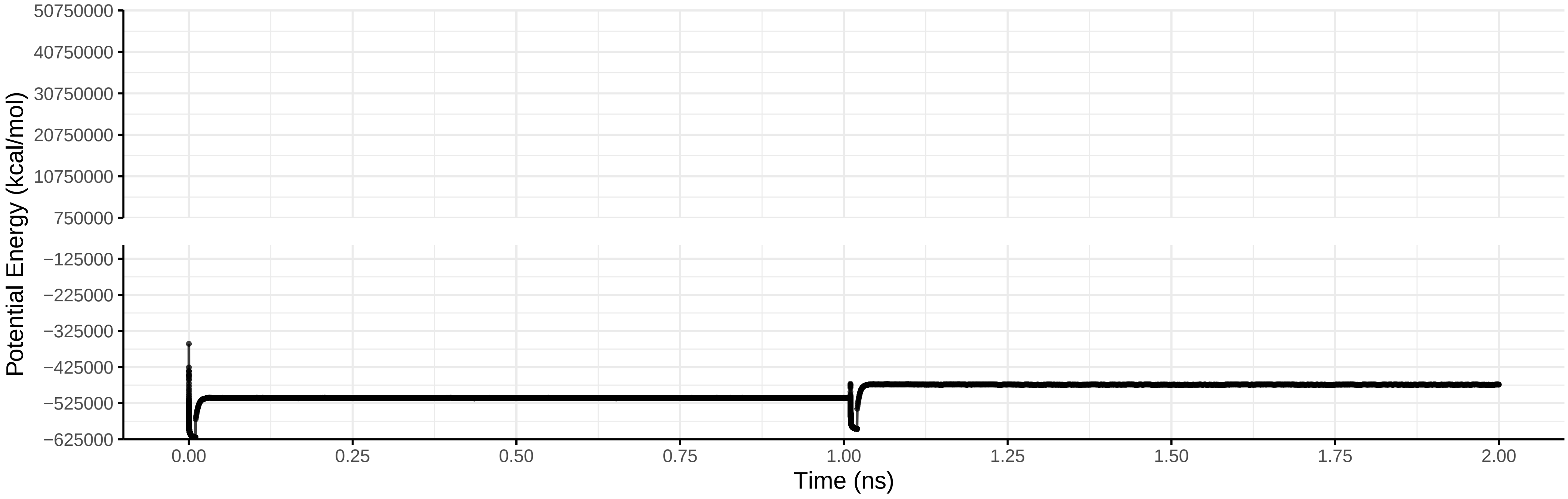

Repeat 1

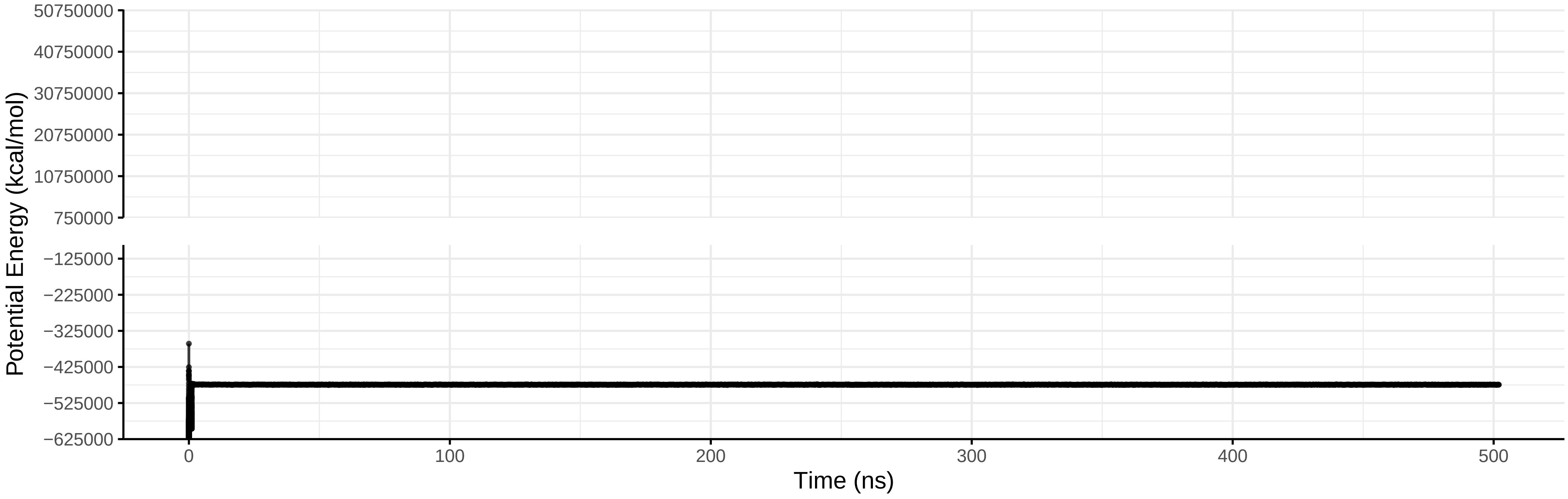

Repeat 2

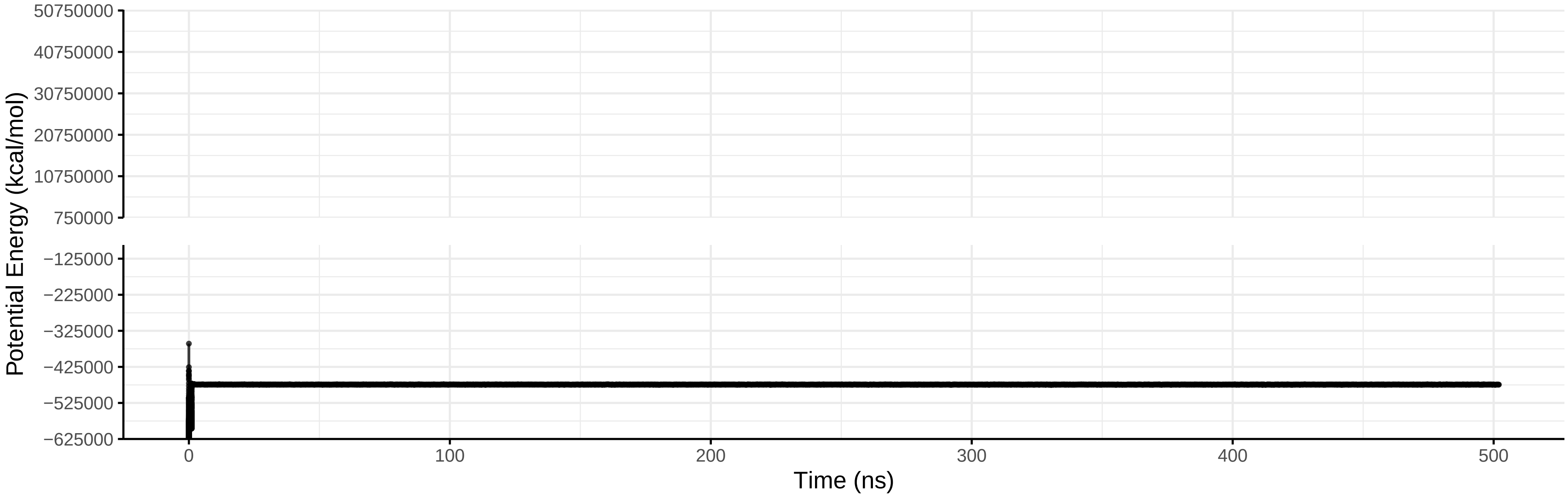

p.Pro389Leu

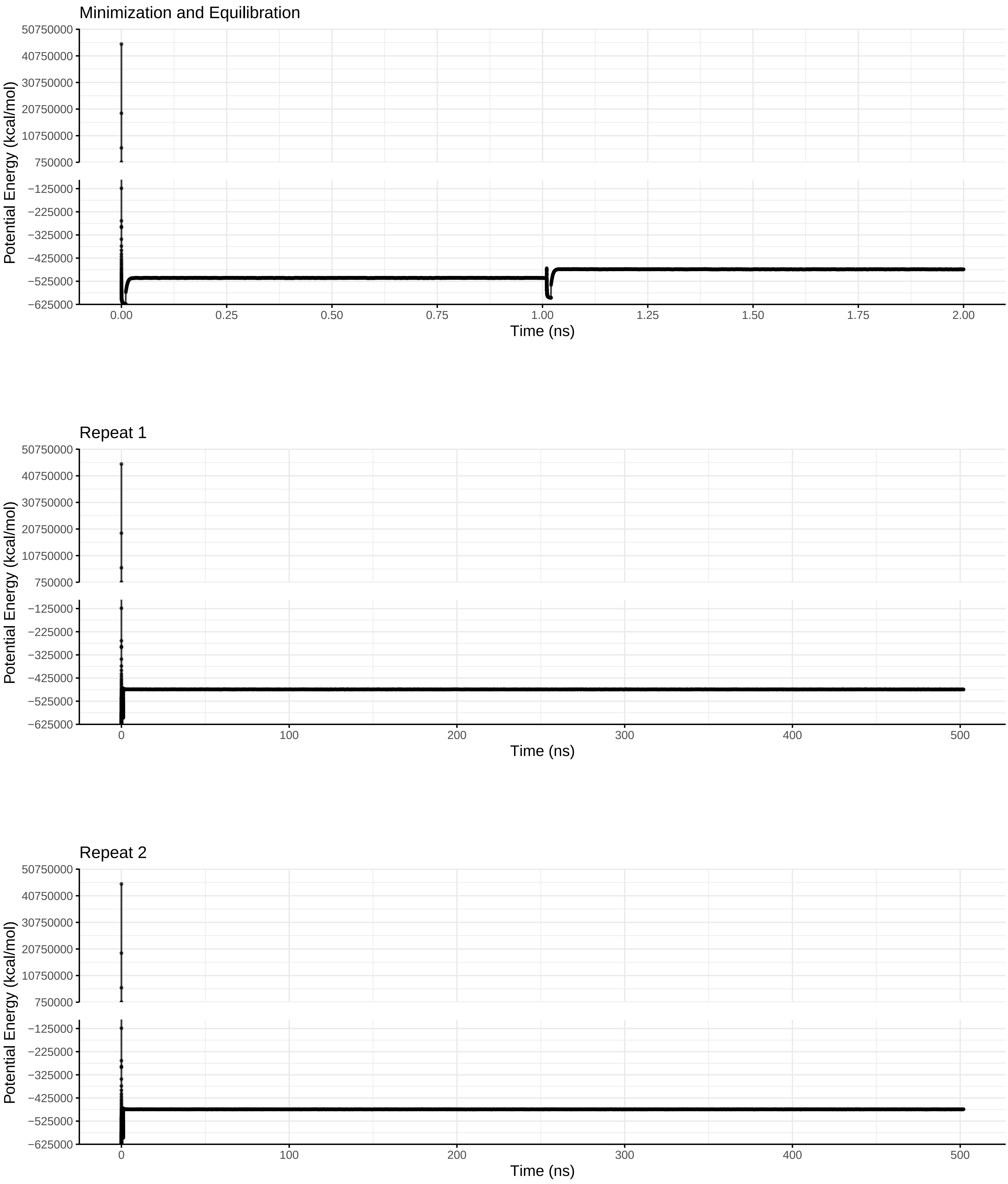
Minimization and Equilibration
Potential Energy (kcal/mol)
50750000
40750000
30750000
20750000
10750000
750000
−125000
−225000
−325000
−425000
−525000
−625000
0.00
0.25
0.50
0.75
1.00
1.25
1.50
1.75
2.00
Time (ns)
Repeat 1
Potential Energy (kcal/mol)
0
100
200
300
400
500
Time (ns)
Repeat 2
Potential Energy (kcal/mol)
0
100
200
300
400
500
Time (ns)

p.Leu430Pro

Minimization and Equilibration

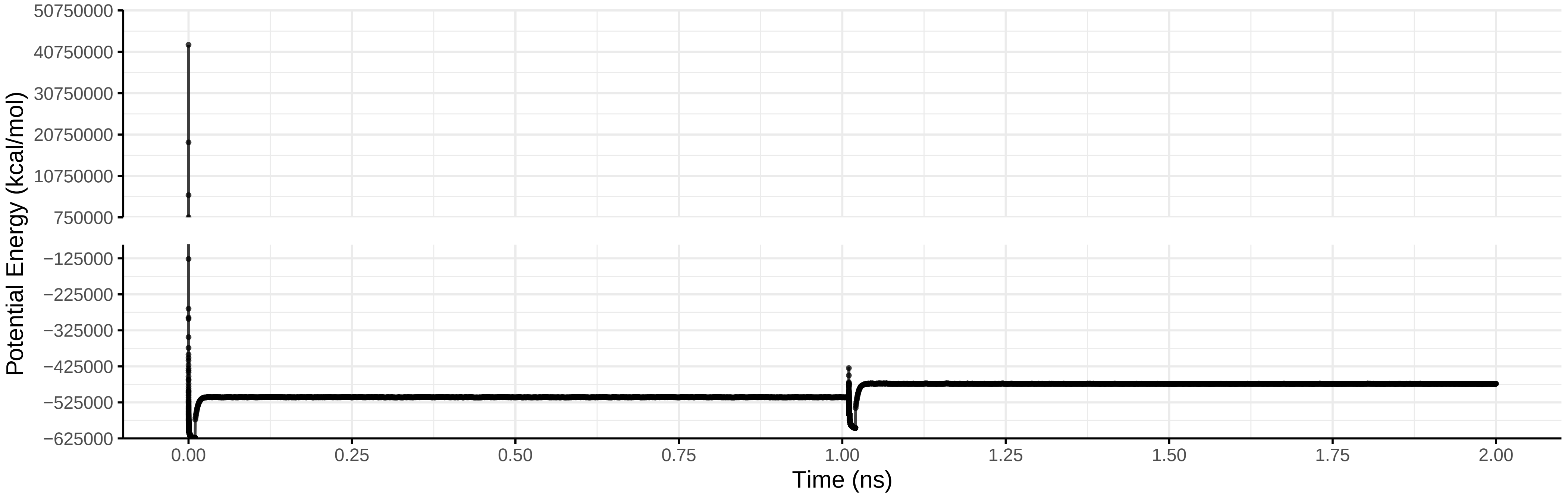

Repeat 1

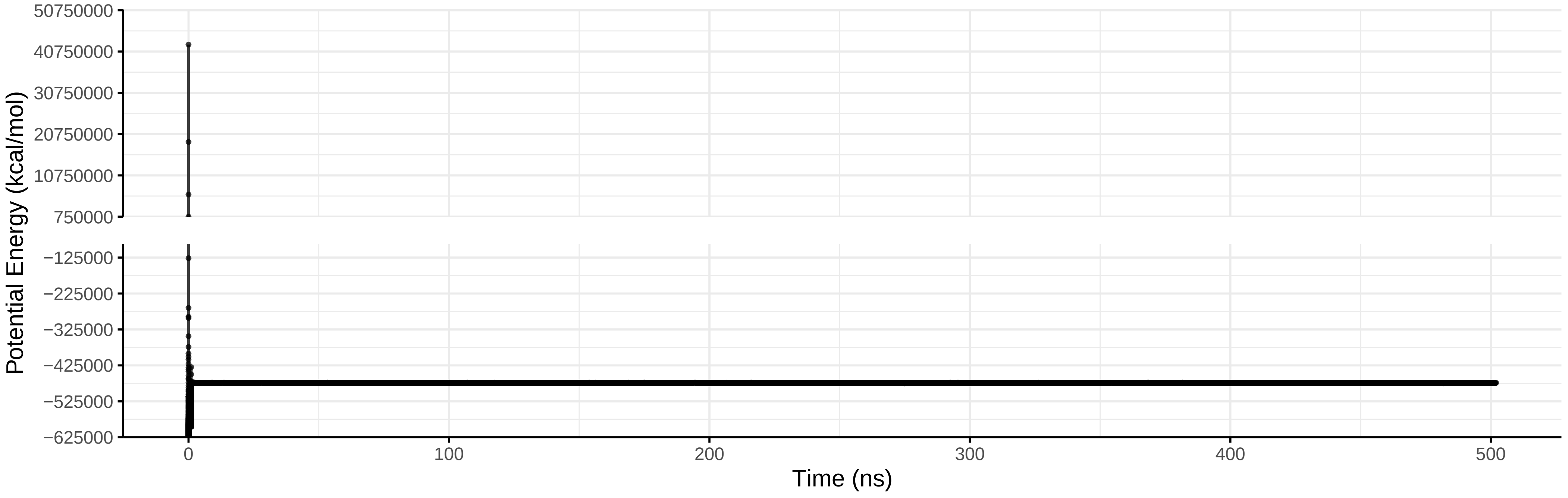

Repeat 2

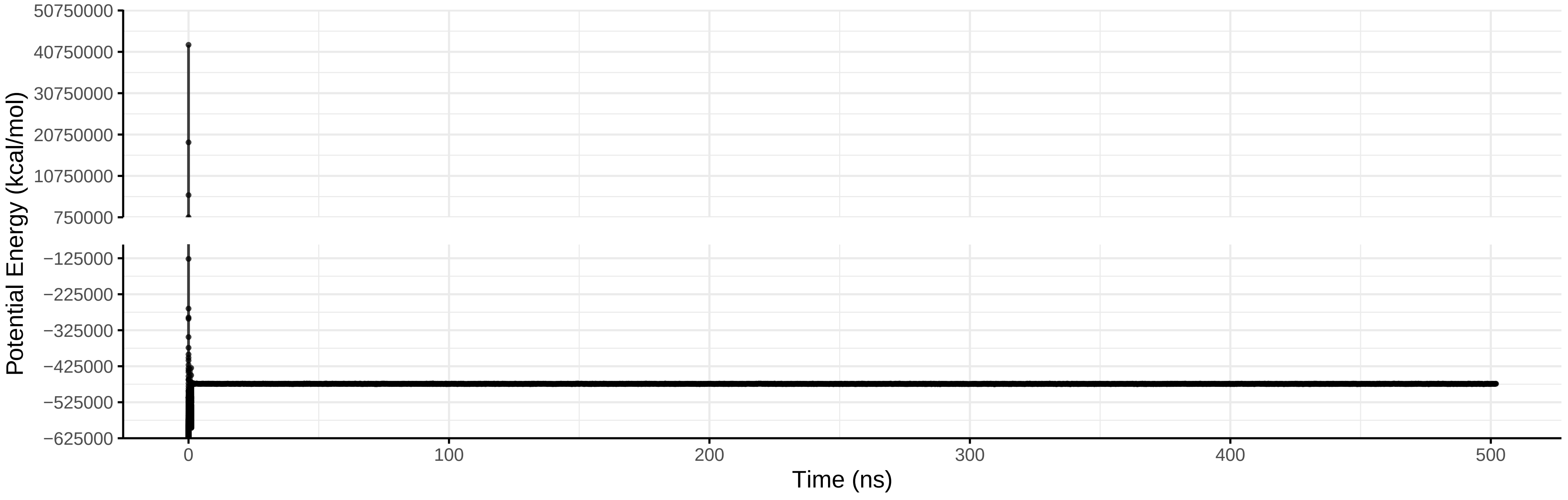

Supplement: Supplemental Information 3 — The energy pattern of each system during the entire simulation procedure. [file peerj-10-12947-s003.pdf]
