# Supplementary material for: Structural analysis of M1AP variants associated with severely impaired spermatogenesis causing male infertility
Source: PeerJ. 2022 Mar 21;10:e12947. doi: 10.7717/peerj.12947 (PMC8944341; doi:10.7717/peerj.12947)
Supplement: Supplemental Information 4 — Alignment of the human M1AP protein to its five orthologs. Yellow stars with labels indicate the positions of the variants examined in this study. [file peerj-10-12947-s004.pdf]

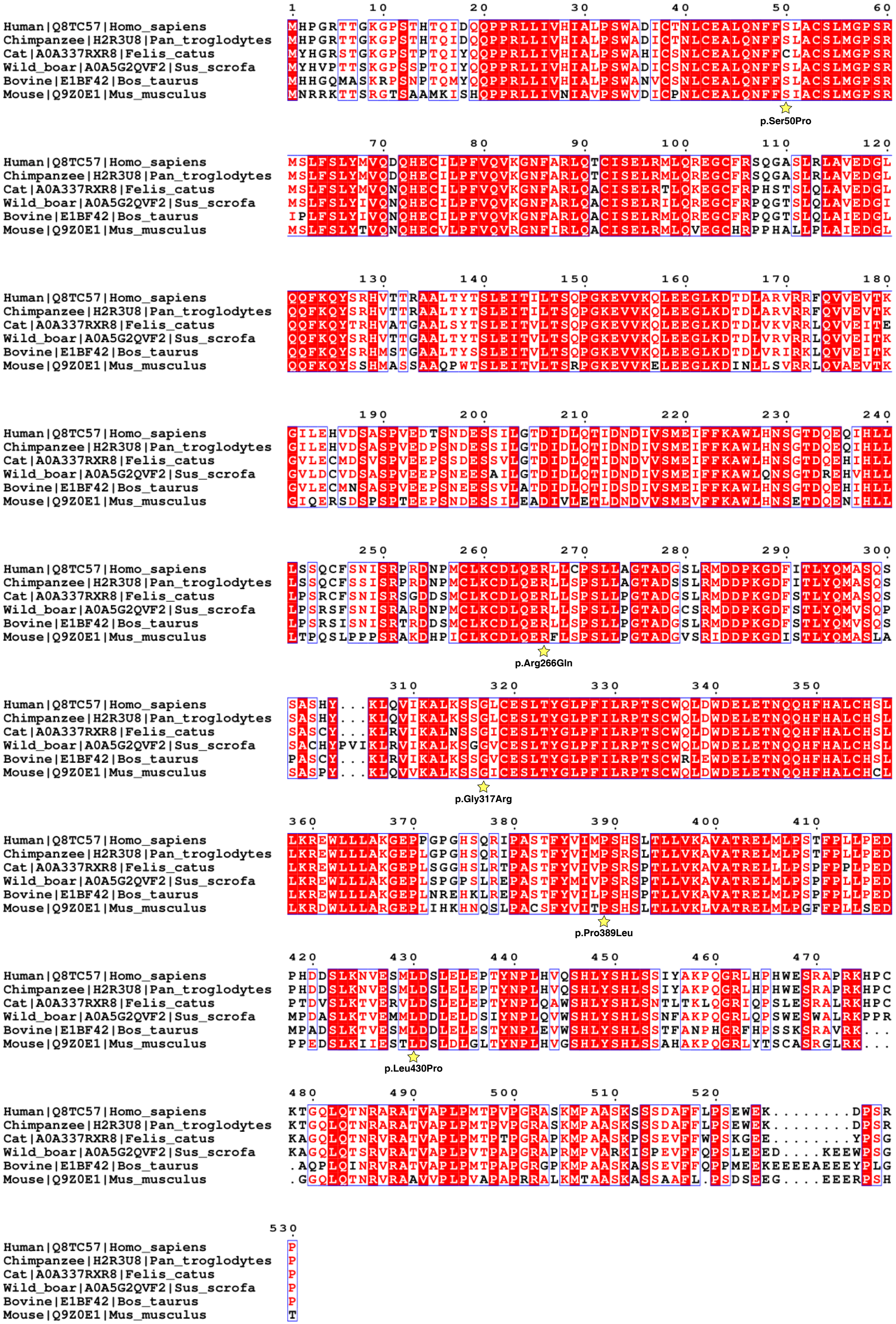
Human|Q8TC57|Homo_sapiens
Chimpanzee|H2R3U8|Pan_troglodytes
Cat|A0A337RXR8|Felis_catus
Wild_boar|A0A5G2QVF2|Sus_scrofa
Bovine|E1BF42|Bos_taurus
Mouse|Q9Z0E1|Mus_musculus
p.Ser50Pro
p.Arg266Gln
p.Gly317Arg
p.Pro389Leu
p.Leu430Pro
