# Supplementary material for: Structural analysis of M1AP variants associated with severely impaired spermatogenesis causing male infertility
Source: PeerJ. 2022 Mar 21;10:e12947. doi: 10.7717/peerj.12947 (PMC8944341; doi:10.7717/peerj.12947)
Supplement: Supplemental Information 8 — Comparison of the local salt bridges between molecular dynamics simulations of wild-type and changed M1AP structures. [file peerj-10-12947-s008.pdf]

p.Ser50Pro

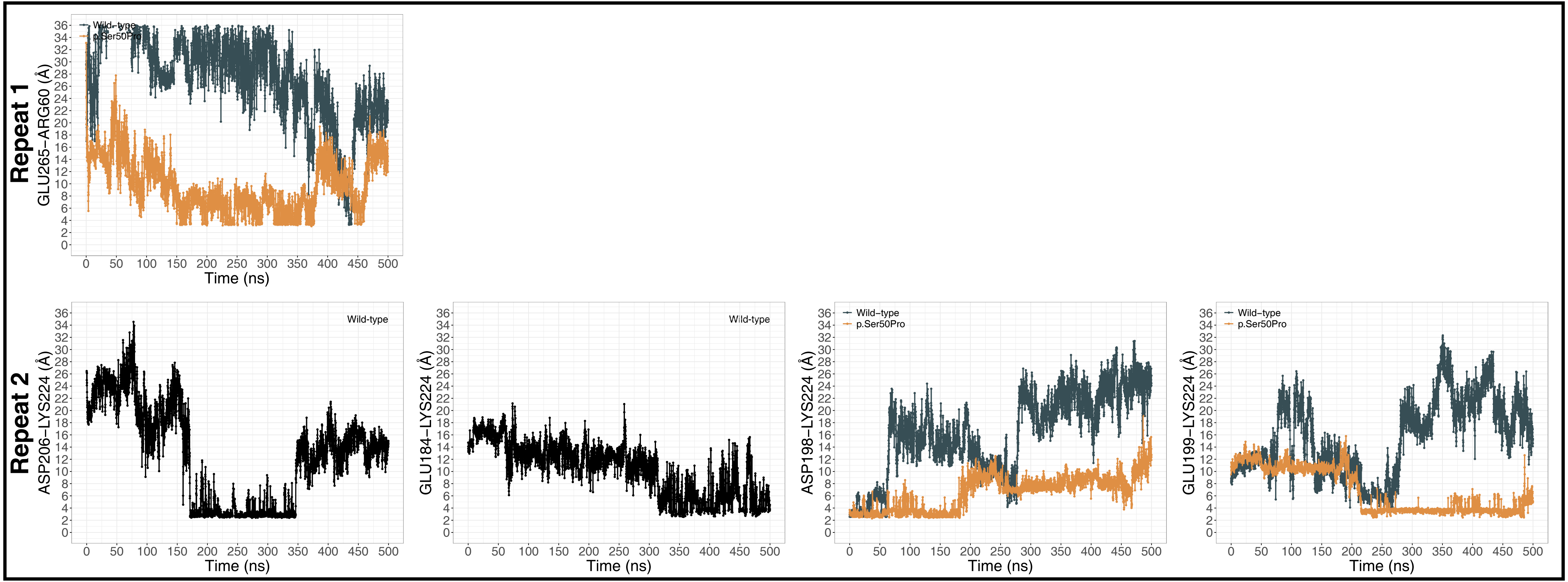

Repeat 1
Repeat 2
Wild-type
p.Ser50Pro
GLU265-ARG60 (Å)
ASP206-LYS224 (Å)
GLU184-LYS224 (Å)
ASP198-LYS224 (Å)
GLU199-LYS224 (Å)
Time (ns)

p.Arg266Gln

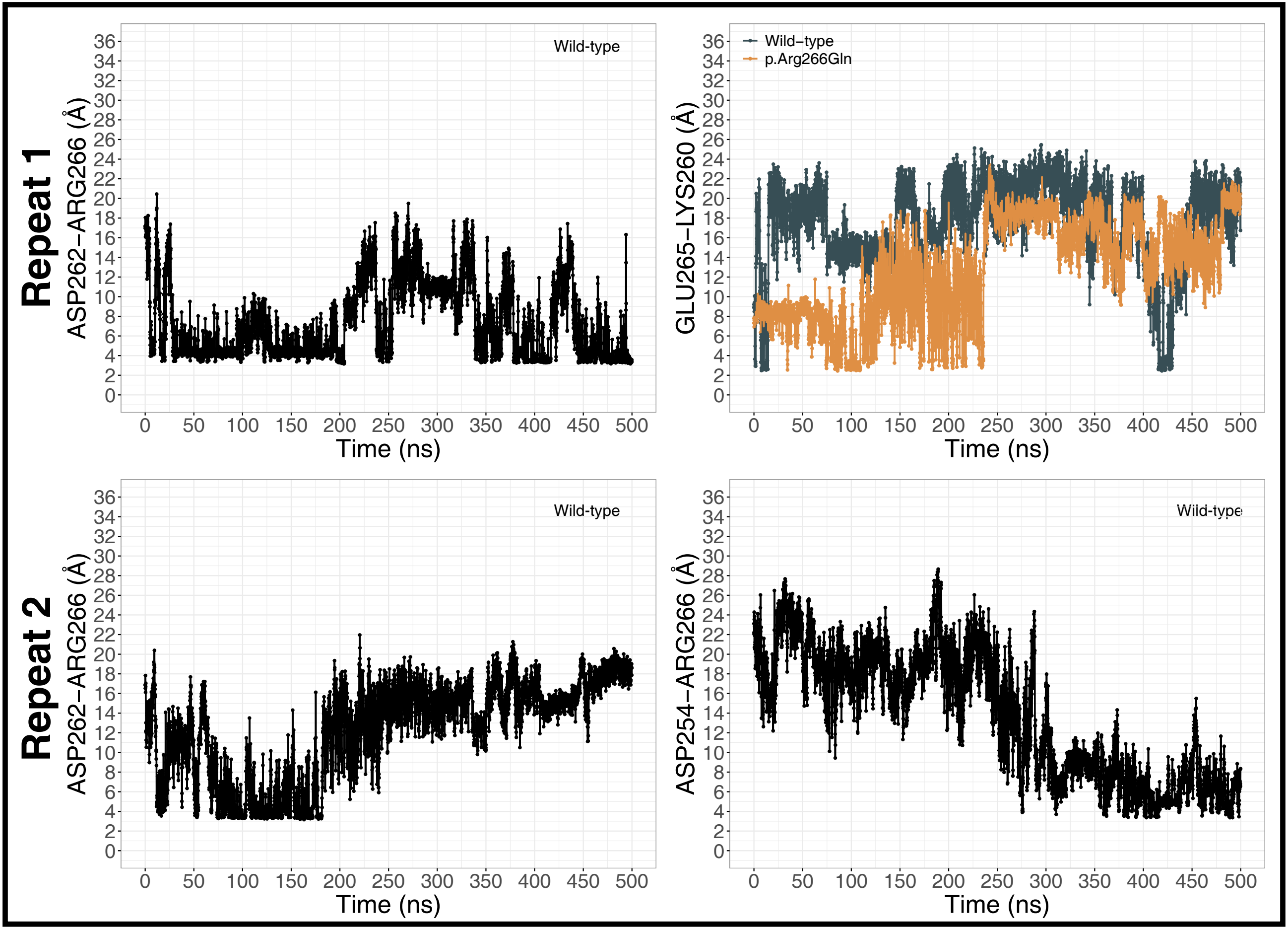

Repeat 1
Repeat 2
ASP262−ARG266 (Å)
GLU265−LYS260 (Å)
ASP254−ARG266 (Å)
Time (ns)
Wild-type
Wild−type
p.Arg266Gln
0
2
4
6
8
10
12
14
16
18
20
22
24
26
28
30
32
34
36
0
50
100
150
200
250
300
350
400
450
500

p.Gly317Arg

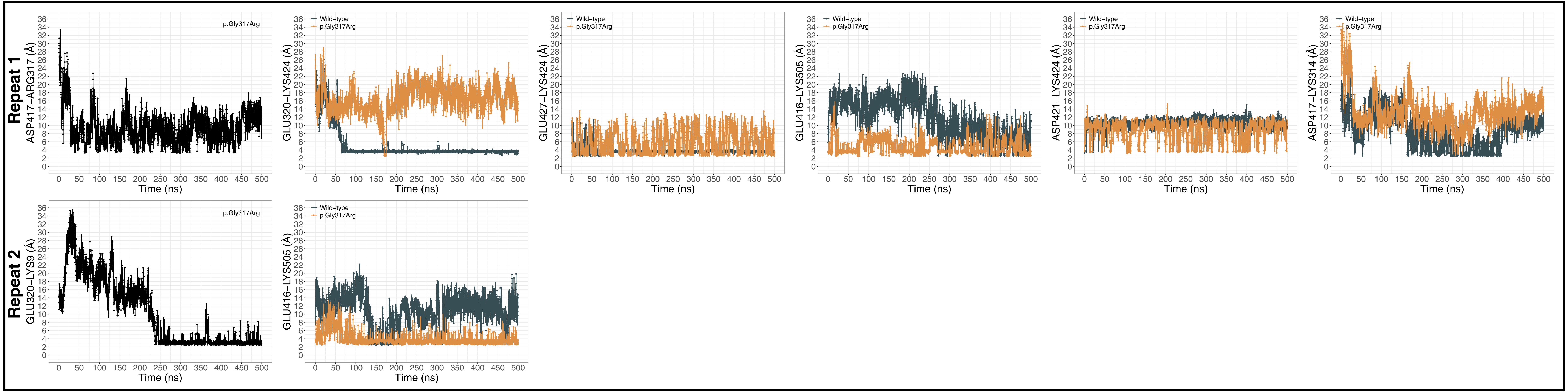

Repeat 1
Repeat 2
p.Gly317Arg
Wild-type
ASP417-ARG317 (Å)
GLU320-LYS424 (Å)
GLU427-LYS424 (Å)
GLU416-LYS505 (Å)
ASP421-LYS424 (Å)
ASP417-LYS314 (Å)
GLU320-LYS9 (Å)
Time (ns)

p.Pro389Leu

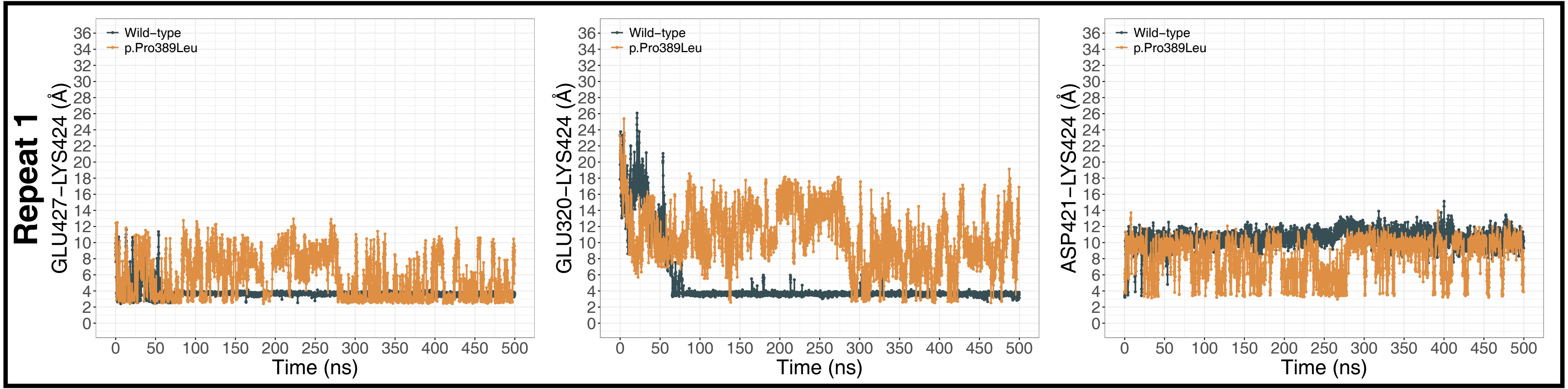
Repeat 1
Wild−type
p.Pro389Leu
GLU427−LYS424 (Å)
GLU320−LYS424 (Å)
ASP421−LYS424 (Å)
Time (ns)
0 2 4 6 8 10 12 14 16 18 20 22 24 26 28 30 32 34 36
0 50 100 150 200 250 300 350 400 450 500

p.Leu430Pro

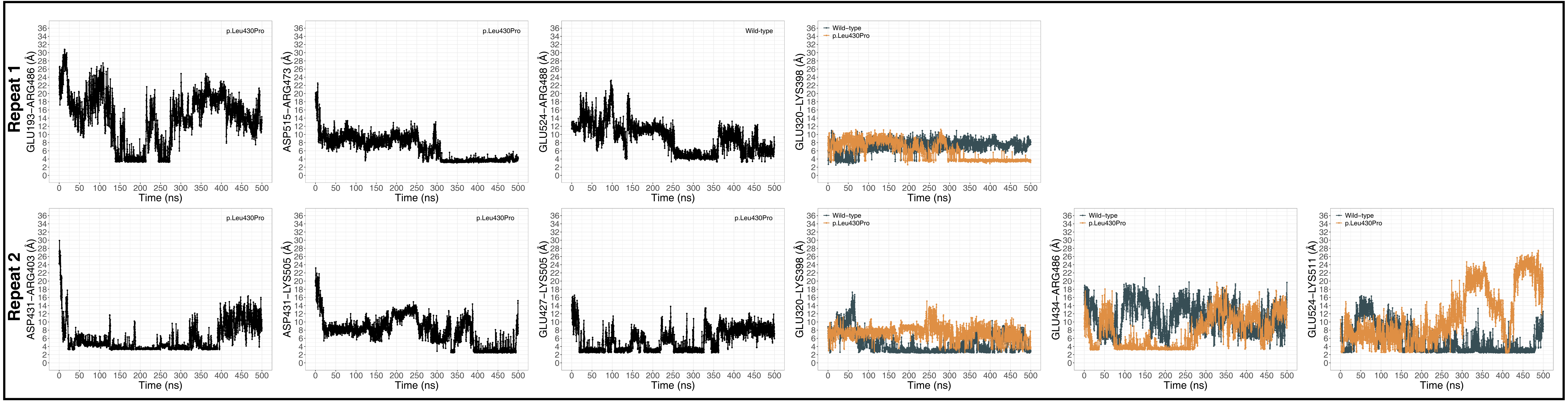
Repeat 1
Repeat 2
p.Leu430Pro
Wild-type
GLU193-ARG486 (Å)
ASP515-ARG473 (Å)
GLU524-ARG488 (Å)
GLU320-LYS398 (Å)
ASP431-ARG403 (Å)
ASP431-LYS505 (Å)
GLU427-LYS505 (Å)
GLU434-ARG486 (Å)
GLU524-LYS511 (Å)
Time (ns)
