# Supplementary material for: Structural analysis of M1AP variants associated with severely impaired spermatogenesis causing male infertility
Source: PeerJ. 2022 Mar 21;10:e12947. doi: 10.7717/peerj.12947 (PMC8944341; doi:10.7717/peerj.12947)
Supplement: Supplemental Information 10 — The methods used to obtain the structures are given with resolution (the smaller the better) and Ramachandran outliers (the smaller the better) indicating the quality of the structures. [file peerj-10-12947-s010.docx]

**Table S1: List of templates assigned for M1AP by structural modeling algorithms.** The methods used to obtain the structures are given with resolution (the smaller the better) and Ramachandran outliers (the smaller the better) indicating the quality of the structures.

| Algorithm | Templates | Method | Resolution (Å) | Ramachandran outliers (%) |
| --- | --- | --- | --- | --- |
| GalaxyWeb | 5Y3R_B, **1JEY_B** | Cryo-EM, **X-ray** | 6.60, **2.50** | 2.4, **0.6** |
| I-TASSER | 1JEQ_A1, **1JEY_B**, 1RS0_A, **2I6Q_A**, 2XWB_F, **3S5H_A**, 5GJV_F, **5Y58_A**, 5Y58_B, **6CIN_A** | X-ray, **X-ray**, X-ray, **X-ray**, X-ray, **X-ray**, Cryo-EM, **X-ray**, X-ray, **X-ray** | 2.70, **2.50**, 2.60, **2.10**, 3.49, **1.60**, 3.60, **2.80**, 2.80, **2.60** | 2.1, **0.6**, 0.6, **0.0**, 1.3, **0.2**, 2.0, **0.0**, 0.0, **0.0** |
| Phyre2 | 1JEY_B, **6FPZ_A**, 1JEY_A, **1JEQ_A**, 1JEY_B1 | X-ray, **X-ray**, X-ray, **X-ray**, X-ray | 2.50, **2.20**, 2.50, **2.70**, 2.50 | 0.6, **0.0**, 0.6, **2.1**, 0.6 |
| PRIMO | 4ACQ_A, **5AH5_A**, 5CK3_B, **5CK4_A**, 6H3V_A | X-ray, **X-ray**, X-ray, **X-ray**, X-ray | 4.30, **2.10**, 3.20, **1.89**, 2.90 | 4.4, **0.3**, 1.5, **0.0**, 0.4 |
| RaptorX | 1T6B_Y, **3N2N_A**, 4WFQ_A, **4HQK_A**, 4C29_A, **1JEQ_A**, 1JEQ_B, **5Y58_B**, 5Y58_A, **5KXF_A**, 3MBO_A, **3USQ_A**, 3LRX_A, **3PM6_A**, 5GVV_A | X-ray, **X-ray**, X-ray, **X-ray**, X-ray, **X-ray**, X-ray, **X-ray**, X-ray, **X-ray**, X-ray, **X-ray**, X-ray, **X-ray**, X-ray | 2.50, **1.80**, 2.40, **2.25**, 2.20, **2.70**, 2.70, **2.80**, 2.80, **2.70**, 3.31, **2.40**, 2.60, **2.20**, 1.95 | 0.5, **0.0**, 0.5, **0.0**, 0.0, **2.1**, 2.1, **0.0**, 0.0, **0.0**, 1.2, **1.2**, 0.5, **0.2**, 0.3 |
| Robetta | 1JEQ_A, **1PCX_A**, 2I6Q_A, **4CNB_B**, 4FX5_A, **4KXF_D**, 5BP4_A, **5CIO_B**, 5O85_C, **5OQJ_A**, 6CIN_A, **6CIN_B**, 6CIN_C, **6CIO_D**, 6HYT_D, **6I2S_A**, 6MG0_A, **6MG0_B**, 6NMI_E, **6R2C_A**, 6R2C_B, **6R2C_C**, 6UIM_A | X-ray, **X-ray**, X-ray, **X-ray**, X-ray, **X-ray**, X-ray, **X-ray**, X-ray, **Cryo-EM**, X-ray, **X-ray**, X-ray, **X-ray**, X-ray, **X-ray**, X-ray, **X-ray**, Cryo-EM, **X-ray**, X-ray, **X-ray**, X-ray | 2.70, **2.50**, 2.10, **1.80**, 1.73, **3.20**, 3.75, **2.50**, 3.40, **4.70**, 2.60, **2.60**, 2.60, **3.00**, 2.33, **2.40**, 6.00, **6.00**, 3.70, **2.09**, 2.09, **2.09**, 2.75 | 2.1, **0.7**, 0.0, **0.9**, 0.0, **0.1**, 1.8, **0.0**, 0.6, **1.2**, 0.0, **0.0**, 0.0, **0.0**, 0.2, **0.1**, 0.1, **0.1**, 0.2, **0.0**, 0.0, **0.0**, 0.5 |
| Robetta-domain^‡^ | 1SHU_X, **2I6Q_A**, 2ODP_A, **2XWJ_I**, 3HRZ_D, **4CN8_A**, 4CN9_A, **4CNB_A**, 4CNB_B, **4FX5_A**, 5NUS_A, **5O85_C**, 5OQJ_A, **5Y58_D**, 6QDX_A, **6QDX_B**, 6QE0_A, **6QT8_A**, 6QTB_A, **6QTA_A**, 6QTB_D | X-ray, **X-ray**, X-ray, **X-ray**, X-ray, **X-ray**, X-ray, **X-ray**, X-ray, **X-ray**, X-ray, **X-ray**, Cryo-EM, **X-ray**, X-ray, **X-ray**, X-ray, **X-ray**, X-ray, **X-ray**, X-ray | 1.50, **2.10**, 1.90, **4.00**, 2.20, **2.45**, 1.90, **1.95**, 1.95, **1.73**, 2.20, **3.40**, 4.70, **2.80**, 2.10, **2.10**, 1.39, **2.33**, 1.89, **1.89**, 1.89 | 0.0, **0.0**, 0.2, **1.3**, 0.2, **0.5**, 0.5, **0.3**, 0.3, **0.0**, 0.0, **0.6**, 1.2, **0.0**, 0.0, **0.0**, 0.0, **0.0**, 0.0, **0.3**, 0.0 |

^†^ In “Templates”, the four characters before the underscore indicate the PDB ID, and the letter after the underscore indicates the protein chain. If there is a number after the chain identifier, it refers to the domain fold in their library.

**^‡^** Robetta-domain automatically used these templates to model amino acids 1-270 of M1AP and used *ab initio* modeling to model amino acids 265-530.
