# Supplementary material for: Structural analysis of M1AP variants associated with severely impaired spermatogenesis causing male infertility
Source: PeerJ. 2022 Mar 21;10:e12947. doi: 10.7717/peerj.12947 (PMC8944341; doi:10.7717/peerj.12947)
Supplement: Supplemental Information 12 — (A) GalaxyWeb, (B) I-TASSER, (C) PRIMO, (D) Robetta and (E) Robetta-domain. [file peerj-10-12947-s012.docx]

**Table S3: Quality assessments of the models built by multiple model generating algorithms from five different algorithms.** (A) GalaxyWeb, (B) I-TASSER, (C) PRIMO, (D) Robetta, and (E) Robetta-domain.

**(A)** Quality assessments of GalaxyWeb’s M1AP models.

| **Model** | **VERIFY3D** | **ERRAT** | **PROVE** | **PROCHECK** | | | | | **WHATCHECK** | | | |
| --- | --- | --- | --- | --- | --- | --- | --- | --- | --- | --- | --- | --- |
|  | Aa% with score ≥ 0.2 | AOQF% | BOPA% | Aa% in MFR | Aa% in AAR | Aa% in GAR | Aa% in DR | G-Factor | # of SCP with Pass | # of SCP with Warning | # of SCP with Error | Overall report |
| **Model 1** | **69.25** | **74.07** | **8.1** | **89.1** | **8.4** | **1.7** | **0.9** | **-0.05** | **26** | **15** | **4** | **Pass** |
| Model 2 | 63.21 | 64.93 | 8.6 | 88.7 | 9.4 | 0.9 | 1.1 | -0.05 | 26 | 15 | 4 | Pass |
| Model 3 | 60.57 | 72.04 | 7.2 | 88.0 | 9.9 | 1.3 | 0.9 | -0.03 | 25 | 16 | 4 | Pass |
| Model 4 | 64.15 | 73.26 | 7.4 | 87.6 | 10.5 | 1.3 | 0.6 | -0.03 | 26 | 15 | 4 | Pass |
| Model 5 | 62.26 | 77.00 | 7.1 | 88.9 | 9.0 | 0.9 | 1.3 | -0.02 | 25 | 16 | 4 | Pass |

^1^ Aa, aa: amino acid. AOQF: average overall quality factor. BOPA: buried outlier protein atoms. MFR: the most favored regions. AAR: additional allowed regions. GAR: generously allowed regions. DR: disallowed regions. SCP: stereochemical properties.

^2^ In VERIFY3D, at least 80% of aa in a protein should have a score ≥ 0.2 in the 3D-1D profile for a good quality.

^3^ In ERRAT, AOQF ≥ 95% indicates a good quality, AOQF around 91% indicates an average quality, and lower AOQF indicates a bad quality.

^4^ In PROVE, BOPA ≤ 1% indicates a good quality, BOPA between 1% and 5% indicates an average quality, and BOPA > 5% indicates a bad quality.

^5^ In the Ramachandran plot analysis of PROCHECK, model has a good quality if overall G-factor is > -0.5 and ≥ 90% of aa in protein are in the most favored regions.

^6^ In WHATCHECK, SCP with Pass refers to a good quality, SCP with Warning indicates an average quality, and SCP with Error refers to a bad quality. Of note, total number of SCP might vary from structure to structure.

^7^ The bold line indicates the best quality model within all models given in the table.

**(B)** Quality assessments of I-TASSER’s M1AP models.

| **Model** | **VERIFY3D** | **ERRAT** | **PROVE** | **PROCHECK** | | | | | **WHATCHECK** | | | |
| --- | --- | --- | --- | --- | --- | --- | --- | --- | --- | --- | --- | --- |
|  | Aa% with score ≥ 0.2 | AOQF% | BOPA% | Aa% in MFR | Aa% in AAR | Aa% in GAR | Aa% in DR | G-Factor | # of SCP with Pass | # of SCP with Warning | # of SCP with Error | Overall report |
| **Model 1** | **47.36** | **90.47** | **7.5** | **60.8** | **31.7** | **4.7** | **2.8** | **-0.77** | **19** | **18** | **10** | **Pass** |
| Model 2 | 54.72 | 73.15 | 7.4 | 62.7 | 27.0 | 6.4 | 3.9 | -0.99 | 16 | 21 | 10 | Pass |
| Model 3 | 31.13 | 83.69 | 7.5 | 67.7 | 24.4 | 4.9 | 3.0 | -0.60 | 21 | 18 | 8 | Pass |
| Model 4 | 53.02 | 76.45 | 6.4 | 57.4 | 36.0 | 3.6 | 3.0 | -0.88 | 18 | 18 | 11 | Pass |
| Model 5 | 50.75 | 71.59 | 6.6 | 50.7 | 35.3 | 9.0 | 4.9 | -1.01 | 18 | 18 | 11 | Pass |

^1^ Aa, aa: amino acid. AOQF: average overall quality factor. BOPA: buried outlier protein atoms. MFR: the most favored regions. AAR: additional allowed regions. GAR: generously allowed regions. DR: disallowed regions. SCP: stereochemical properties.

^2^ In VERIFY3D, at least 80% of aa in a protein should have a score ≥ 0.2 in the 3D-1D profile for a good quality.

^3^ In ERRAT, AOQF ≥ 95% indicates a good quality, AOQF around 91% indicates an average quality, and lower AOQF indicates a bad quality.

^4^ In PROVE, BOPA ≤ 1% indicates a good quality, BOPA between 1% and 5% indicates an average quality, and BOPA > 5% indicates a bad quality.

^5^ In the Ramachandran plot analysis of PROCHECK, model has a good quality if overall G-factor is > -0.5 and ≥ 90% of aa in protein are in the most favored regions.

^6^ In WHATCHECK, SCP with Pass refers to a good quality, SCP with Warning indicates an average quality, and SCP with Error refers to a bad quality. Of note, total number of SCP might vary from structure to structure.

^7^ The bold line indicates the best quality model within all models given in the table.

**(C)** Quality assessments of PRIMO’s M1AP models.

| **Model** | **VERIFY3D** | **ERRAT** | **PROVE** | **PROCHECK** | | | | | **WHATCHECK** | | | |
| --- | --- | --- | --- | --- | --- | --- | --- | --- | --- | --- | --- | --- |
|  | Aa% with score ≥ 0.2 | AOQF% | BOPA% | Aa% in MFR | Aa% in AAR | Aa% in GAR | Aa% in DR | G-Factor | # of SCP with Pass | # of SCP with Warning | # of SCP with Error | Overall report |
| **Model 1** | **39.66** | **1.35** | **33.4** | **58.4** | **25.1** | **9.2** | **7.3** | **-1.41** | **23** | **15** | **8** | **Pass** |
| Model 2 | 38.33 | 1.16 | 29.5 | 56.2 | 30.0 | 7.5 | 6.2 | -1.40 | 22 | 16 | 8 | Pass |
| Model 3 | 37.00 | 0.19 | 37.9 | 59.9 | 25.8 | 8.4 | 6.0 | -1.61 | 22 | 15 | 9 | Pass |
| Model 4 | 41.75 | 2.31 | 30.5 | 59.4 | 25.3 | 8.4 | 6.9 | -1.30 | 22 | 16 | 8 | Pass |

^1^ Aa, aa: amino acid. AOQF: average overall quality factor. BOPA: buried outlier protein atoms. MFR: the most favored regions. AAR: additional allowed regions. GAR: generously allowed regions. DR: disallowed regions. SCP: stereochemical properties.

^2^ In VERIFY3D, at least 80% of aa in a protein should have a score ≥ 0.2 in the 3D-1D profile for a good quality.

^3^ In ERRAT, AOQF ≥ 95% indicates a good quality, AOQF around 91% indicates an average quality, and lower AOQF indicates a bad quality.

^4^ In PROVE, BOPA ≤ 1% indicates a good quality, BOPA between 1% and 5% indicates an average quality, and BOPA > 5% indicates a bad quality.

^5^ In the Ramachandran plot analysis of PROCHECK, model has a good quality if overall G-factor is > -0.5 and ≥ 90% of aa in protein are in the most favored regions.

^6^ In WHATCHECK, SCP with Pass refers to a good quality, SCP with Warning indicates an average quality, and SCP with Error refers to a bad quality. Of note, total number of SCP might vary from structure to structure.

^7^ The bold line indicates the best quality model within all models given in the table.

**(D)** Quality assessments of Robetta’s M1AP models.

| **Model** | **VERIFY3D** | **ERRAT** | **PROVE** | **PROCHECK** | | | | | **WHATCHECK** | | | |
| --- | --- | --- | --- | --- | --- | --- | --- | --- | --- | --- | --- | --- |
|  | Aa% with score ≥ 0.2 | AOQF% | BOPA% | Aa% in MFR | Aa% in AAR | Aa% in GAR | Aa% in DR | G-Factor | # of SCP with Pass | # of SCP with Warning | # of SCP with Error | Overall report |
| Model 1 | 89.25 | 87.52 | 5.8 | 83.5 | 14.8 | 1.1 | 0.6 | 0.13 | 26 | 14 | 7 | Pass |
| Model 2 | 80.19 | 86.35 | 5.8 | 82.4 | 16.3 | 0.6 | 0.6 | 0.05 | 26 | 15 | 6 | Pass |
| Model 3 | 63.58 | 85.85 | 5.8 | 79.7 | 17.6 | 2.6 | 0.2 | 0.08 | 26 | 16 | 5 | Pass |
| Model 4 | 64.72 | 80.47 | 5.6 | 80.7 | 17.1 | 1.1 | 1.1 | 0.02 | 25 | 15 | 7 | Pass |
| **Model 5** | **76.23** | **95.22** | **4.8** | **83.9** | **15.6** | **0.2** | **0.2** | **0.11** | **27** | **14** | **6** | **Pass** |

^1^ Aa, aa: amino acid. AOQF: average overall quality factor. BOPA: buried outlier protein atoms. MFR: the most favored regions. AAR: additional allowed regions. GAR: generously allowed regions. DR: disallowed regions. SCP: stereochemical properties.

^2^ In VERIFY3D, at least 80% of aa in a protein should have a score ≥ 0.2 in the 3D-1D profile for a good quality.

^3^ In ERRAT, AOQF ≥ 95% indicates a good quality, AOQF around 91% indicates an average quality, and lower AOQF indicates a bad quality.

^4^ In PROVE, BOPA ≤ 1% indicates a good quality, BOPA between 1% and 5% indicates an average quality, and BOPA > 5% indicates a bad quality.

^5^ In the Ramachandran plot analysis of PROCHECK, model has a good quality if overall G-factor is > -0.5 and ≥ 90% of aa in protein are in the most favored regions.

^6^ In WHATCHECK, SCP with Pass refers to a good quality, SCP with Warning indicates an average quality, and SCP with Error refers to a bad quality. Of note, total number of SCP might vary from structure to structure.

^7^ The bold line indicates the best quality model within all models given in the table.

**(E)** Quality assessments of Robetta-domain’s M1AP models.

| **Model** | **VERIFY3D** | **ERRAT** | **PROVE** | **PROCHECK** | | | | | **WHATCHECK** | | | |
| --- | --- | --- | --- | --- | --- | --- | --- | --- | --- | --- | --- | --- |
|  | Aa% with score ≥ 0.2 | AOQF% | BOPA% | Aa% in MFR | Aa% in AAR | Aa% in GAR | Aa% in DR | G-Factor | # of SCP with Pass | # of SCP with Warning | # of SCP with Error | Overall report |
| **Model 1** | **89.06** | **88.24** | **4.4** | **85.2** | **13.9** | **0.4** | **0.4** | **0.20** | **27** | **15** | **5** | **Pass** |
| Model 2 | 75.28 | 89.96 | 5.3 | 84.8 | 14.1 | 0.6 | 0.4 | 0.16 | 27 | 14 | 6 | Pass |
| Model 3 | 86.42 | 85.85 | 5.6 | 87.2 | 11.1 | 0.6 | 1.1 | 0.18 | 26 | 15 | 6 | Pass |
| Model 4 | 78.11 | 90.84 | 4.1 | 83.9 | 14.8 | 0.6 | 0.6 | 0.18 | 25 | 16 | 6 | Pass |
| Model 5 | 79.81 | 90.06 | 4.0 | 86.7 | 11.8 | 0.9 | 0.6 | 0.22 | 26 | 15 | 6 | Pass |

^1^ Aa, aa: amino acid. AOQF: average overall quality factor. BOPA: buried outlier protein atoms. MFR: the most favored regions. AAR: additional allowed regions. GAR: generously allowed regions. DR: disallowed regions. SCP: stereochemical properties.

^2^ In VERIFY3D, at least 80% of aa in a protein should have a score ≥ 0.2 in the 3D-1D profile for a good quality.

^3^ In ERRAT, AOQF ≥ 95% indicates a good quality, AOQF around 91% indicates an average quality, and lower AOQF indicates a bad quality.

^4^ In PROVE, BOPA ≤ 1% indicates a good quality, BOPA between 1% and 5% indicates an average quality, and BOPA > 5% indicates a bad quality.

^5^ In the Ramachandran plot analysis of PROCHECK, model has a good quality if overall G-factor is > -0.5 and ≥ 90% of aa in protein are in the most favored regions.

^6^ In WHATCHECK, SCP with Pass refers to a good quality, SCP with Warning indicates an average quality, and SCP with Error refers to a bad quality. Of note, total number of SCP might vary from structure to structure.

^7^ The bold line indicates the best quality model within all models given in the table.
